# Biosynthesis of barley wax β-diketones: a type-III polyketide synthase condensing two fatty acyl units

**DOI:** 10.1038/s41467-023-42917-9

**Published:** 2023-11-10

**Authors:** Yulin Sun, Alberto Ruiz Orduna, Zhonghang Zhang, Sarah J. Feakins, Reinhard Jetter

**Affiliations:** 1 Department of Botany, University of British Columbia, Vancouver, BC V6T 1Z4 Canada; 2 Department of Chemistry, University of British Columbia, Vancouver, BC V6T 1Z1 Canada; 3 Department of Earth Sciences, University of Southern California, 3651 Trousdale Pkwy, Los Angeles, CA 90089 USA

**Keywords:** Secondary metabolism, Kinases, Waxes

## Abstract

The surface coatings of cereal plants are dominated by waxy β-diketones crucial for drought resistance and, therefore, grain yield. Here, barley (*Hordeum vulgare*) wax analyses reveal β-diketone and associated 2-alkanol ester profiles suggesting a common C_16_ 3-ketoacid precursor. Isotope analysis further shows that the major (C_31_) diketone is synthesized from two plastidial C_16_ acyl units. Previous studies identified a gene cluster encoding enzymes responsible for β-diketone formation in barley, but left their biochemical functions unknown. Various assays now characterize one of these enzymes as a thioesterase producing long-chain (mainly C_16_) 3-ketoacids, and another one as a polyketide synthase (PKS) condensing the 3-ketoacids with long-chain (mainly C_16_) acyl-CoAs into β-diketones. The two enzymes are localized to the plastids and Endoplasmic Reticulum (ER), respectively, implying substrate transfer between these two sub-cellular compartments. Overall, our findings define a two-step pathway involving an unprecedented PKS reaction leading directly to the β-diketone products.

## Abstract (web summary)

Barley plants coat their organs with waxy diketones to protect against late-summer droughts. These diketones are formed by two enzymes, one diverting common fatty acids from normal metabolism and the other one linking two fatty acid units together.

## Introduction

Plants must seal the vast surfaces of their above-ground organs against water loss and pathogens^1^. They achieve this crucial eco-physiological function with waxes formed in their epidermis and deposited as a thin layer on the surface^1,2^. The waxes are complex mixtures of aliphatic compounds mostly derived from fatty acid metabolism, with aliphatic chains typically containing 24–34 carbons^3^. Most wax compounds have oxygen functional groups, predominantly at one chain end but also on mid-chain carbons. The composition of wax mixtures varies substantially between plant species and organs, leading to characteristic distributions of compound classes and chain lengths within each of them^3,4^. The common wax compounds are synthesized in three stages, involving (i) formation of C_16_ fatty acyls by plastidial fatty acid synthases (FASs); (ii) further elongation to very-long-chain (VLC) acyl-CoAs by fatty acid elongases (FAEs) in the endoplasmic reticulum (ER); (iii) modification of the carboxyl headgroups on pathways involving either reduction to alcohols or decarbonylation to alkanes^2^.

**Fig. 1:**
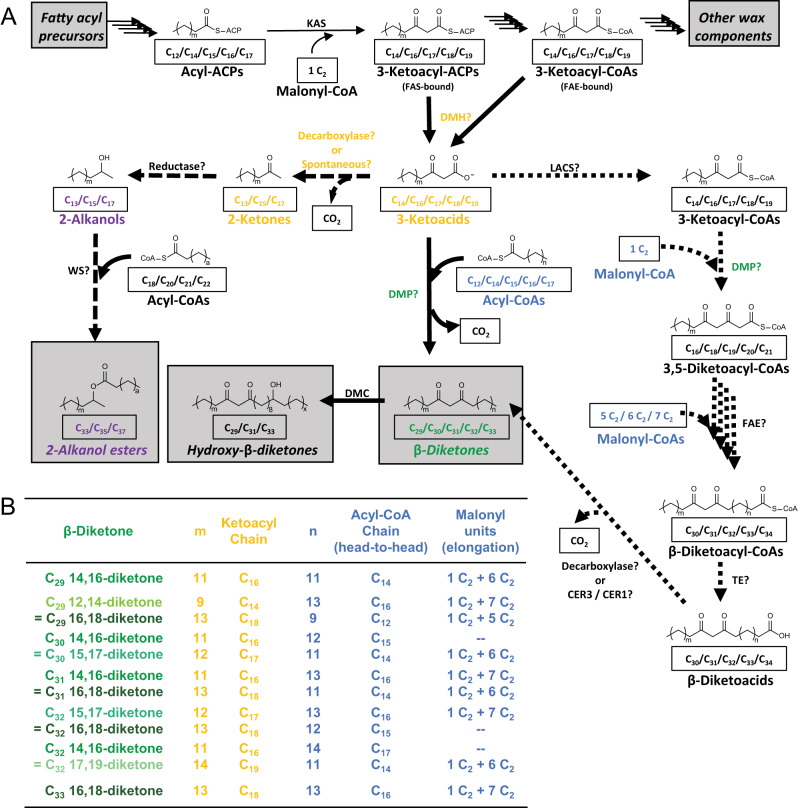
Proposed β-diketone-forming pathways in barley. **A** Pathways leading from acyl precursors (top left gray box) to various wax components (other gray boxes). Common wax components are formed by acyl-Acyl Carrier Protein (ACP) and acyl-CoA elongation (top row). The branch pathways leading to β-diketones and associated 2-alkanol esters (bottom half of scheme) proceed via central 3-ketoacid intermediates: diketone metabolism hydrolase (DMH) intercepts 3-ketoacyl-ACPs of plastidial Fatty Acid Synthase (FAS) or 3-ketoacyl-CoA intermediates of ER-bound Fatty Acid Elongase (FAE). The 3-ketoacids are either converted into 2-alkanol esters (left side; long-dashed arrows) or into β-diketones (right side). Two reaction paths from 3-ketoacids to β-diketones are feasible: (i) The elongation hypothesis (short-dashed arrows) involves activation by a Long-Chain Acyl-CoA Synthetase (LACS), condensation with a C_2_ unit (from malonyl-CoA) by diketone metabolism polyketide synthase (DMP), further elongations catalyzed by FAE(s), and head group removal either by a thioesterase (TE) and a decarboxylase or by CER3/CER1-like enzymes. (ii) Alternatively, the head-to-head condensation hypothesis (solid arrows) predicts that DMP condenses fatty acyl-CoA starters and 3-ketoacid extenders. A diketone metabolism cytochrome-P450 (DMC) enzyme then likely hydroxylates β-diketones to produce hydroxy-β-diketones. **B** List of β-diketones in barley waxes, with isomers color-coded by diketo-group position. For isomers with n ≠ m two alternative names counting from either terminus are given. The chain length of the ketoacyl generating each β-diketone is given in orange. The acyl-CoA chain length required for head-to-head condensation to each β-diketone and the number of malonyl extender units required for the alternative elongation pathway are given in blue. Dashes indicate β-diketone isomers which cannot be synthesized by elongation. KAS β-ketoacyl-ACP synthase, WS wax ester synthase, CER3 ECERIFERUM 3, CER1 ECERIFERUM 1.

Many plant species accumulate specialty compounds in their surface waxes, frequently in the form of mid-chain β-diketones. Characteristic VLC β-diketones are found in diverse dicots (e.g., Myrtaceae, Buxaceae, Ericaceae, Caryophyllaceae, and Asteraceae)^5–10^ and monocots (e.g., *Hosta* and *Vanilla* spp.*)*^11,12^. The wax β-diketones are especially widespread among the Poaceae^10^, including crops such as wheat (*Triticum durum*, *T. aestivum*)^13–15^, barley (*Hordeum vulgare*)^8,16^, and rye (*Secale cereale*)^17^. For example, barley waxes contain predominantly C_31_ 14,16-diketone, together with minor amounts of C_33_ and C_29_ diketones^8^. In many species, β-diketones are accompanied by esters of 2-alkanols thought to be side products of β-diketone formation (Fig. 1A)^10,18^.

β-Diketones are produced in late vegetative and reproductive growth stages in Poaceae crops, especially in the waxes covering upper leaf sheaths, flag leaves and spikes^15,16,19,20^. There, they are among the most dynamically regulated wax components during drought conditions^21^, associated with high water use efficiency and light reflectance^22^. Therefore, β-diketones are crucial for maintaining grain yield under stress, and their accumulation is a favorable agronomic trait that has been highly selected in crop breeding^23^. However, the mechanism underlying β-diketone formation is only partially understood.

Early biochemical attempts to address β-diketone synthesis in barley with isotopic tracing suggested 3-ketoacids as key intermediates en route to C_31_ 14,16-diketone (Fig. 1A and Supplementary Fig. S1)^8,24–26^, but lacked definitive evidence for this assumption. Recently, genetic studies identified metabolic gene clusters involved in β-diketone formation in both wheat and barley, each defined by the presence of three key genes. These genes were predicted to encode a hydrolase/carboxylesterase (named diketone metabolism hydrolase, DMH), a type-III polyketide synthase (PKS) (named diketone metabolism PKS, DMP) and a cytochrome-P450 oxidase (named diketone metabolism CYP450, DMC)^19,20^. In the process, the barley orthologs were recognized as the *Cer-q*, *Cer-c* and *Cer-u* genes described in earlier genetic studies, respectively^20,27^. Earlier mutant analyses had shown that the barley Cer-q/HvDMH and Cer-c/HvDMP enzymes were both required for β-diketone formation, and more recent VIGS experiments confirmed the in planta function of the corresponding enzymes also in wheat^19,20^ Thus, all previous evidence taken together suggested that DMH catalyzes the first step of β-diketone formation leading to the 3-ketoacid intermediate, and preliminary analyses confirmed that barley DMH may indeed intercept fatty acyl elongation intermediates to form 3-ketoacids^19^. While this provided first evidence for the enzymatic reaction leading to the key intermediate, it did not provide information on the later steps in the pathway.

Two fundamentally different mechanisms for the DMP-catalyzed formation of the diketo group must be considered (Fig. 1A and Supplementary Fig. S1). On the one hand, DMP may catalyze the condensation of 3-ketoacyl-CoA starters and malonyl-CoA extenders^10^, resembling the activities of most PKSs^28^. The resulting 3,5-diketoacyl-CoA would require further elongation, likely by FAE-type elongase(s), and head group modification, similar to the pathways leading to common wax compounds. The major β-diketone of barley and wheat waxes, C_31_ 14,16-diketone, may thus be formed via C_16_ 3-ketoacid or C_18_ 3-ketoacid as central pathway intermediate. However, both the nature of the intermediate and the enzymes modifying it in later steps of the predicted pathway remained elusive, despite the characterization of numerous barley β-diketone-deficient mutants. On the other hand, DMP could condense the 3-ketoacid intermediates with common fatty acyl-CoAs to directly form β-diketone products. Such a head-to-head condensation of two pre-formed hydrocarbon chains had been proposed early on^29^, but various wax formation pathways were since shown to involve incremental build-up of hydrocarbon chains^2^. This second hypothesis for β-diketone biosynthesis thus diverges substantially from previously known wax formation mechanisms. Furthermore, it also invokes DMP substrates very different from those of most plant PKSs, which prefer aromatic starters and malonyl extenders^28,30,31^. However, it had been noted that the wheat DMP protein shares sequence similarity with *Curcuma longa* curcumin synthase (CURS)^19^, a PKS enzyme known to use an aromatic ketoacid extender for head-to-head condensation with a second aromatic acyl^32^. Here, we aimed to revisit both these fundamental models for hydrocarbon chain formation and to elucidate the overall biosynthetic pathway leading to β-diketones in barley.

## Results

To elucidate the β-diketone biosynthesis pathway, we aimed to provide both chemical evidence defining the major pathway intermediates and biochemical evidence characterizing the key enzymes involved.

### Homolog and isomer distributions of β-diketones and 2-alkanol esters indicate the major pathway intermediate

In a first set of experiments, we sought to identify all β-diketone homolog and isomer structures accumulating in barley cv. Morex spike wax. To this end, the wax mixture was separated by thin-layer chromatography (TLC), and the fraction containing the β-diketones (R_f_ 0.56) was analyzed by gas chromatography-mass spectrometry (GC-MS). The β-diketones were identified by characteristic base peaks *m/z* 100^33^, while molecular ions and [M-18]^+^ ions enabled the assignment of overall chain lengths C_29_ to C_33_ (Supplementary Fig. S2). Positional isomers for each homolog were assigned based on prominent fingerprint fragments, leading to the identification of C_29_ 12,14- and 14,16-diketone, C_31_ 14,16-diketone and C_33_ 16,18-diketone structures reported before^8^. In addition, novel even-numbered homologs were identified as C_30_ 14,16-diketone and a mixture of C_32_ 14,16- and C_32_ 15,17-diketones. Analysis of the GC retention times showed that the even-numbered β-diketone homologs had straight hydrocarbon backbones devoid of methyl branches (Supplementary Fig. S3)﻿, implying that one of their acyl moieties must be formed from an unbranched, odd-numbered starter of de novo fatty acid synthesis.

**Fig. 2:**
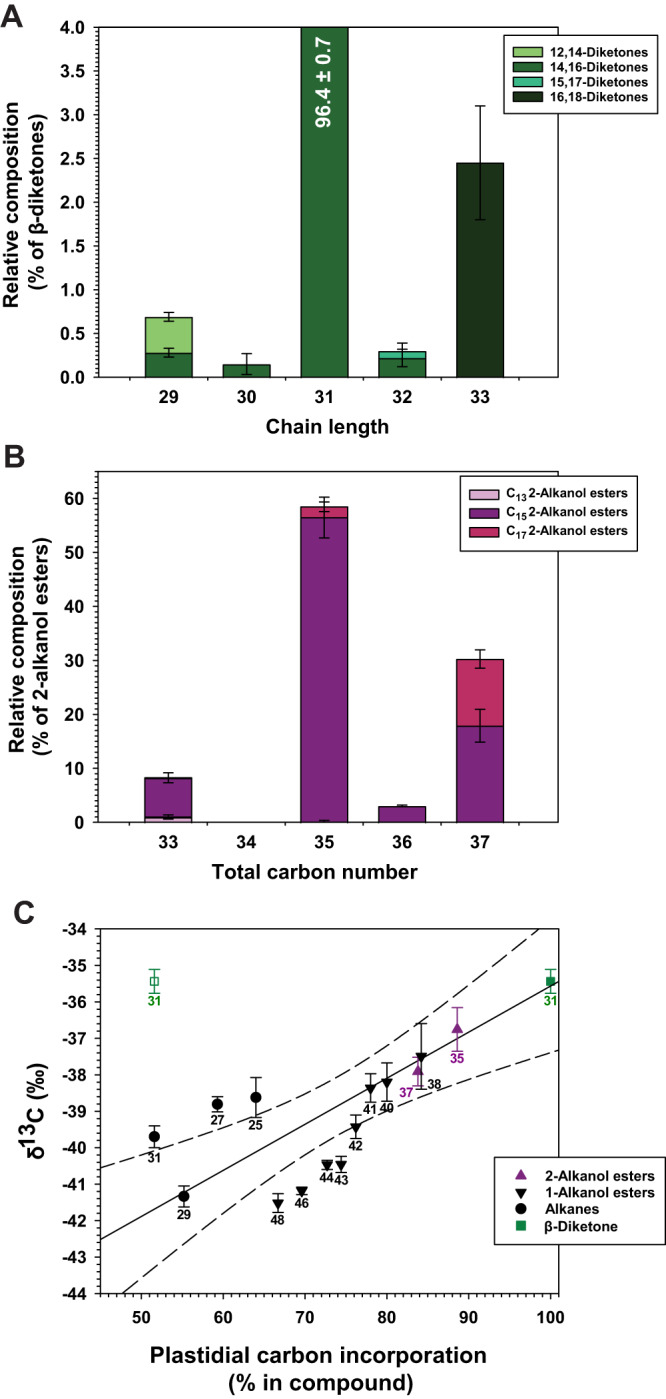
Chemical characterization of the β-diketones and associated 2-alkanol esters in the wax mixture covering barley cv. Morex spikes. **A** Relative amounts of β-diketone homologs (determined by GC-MS analysis of the characteristic fragment *m/*z 100) and isomers (determined using characteristic α-fragments). **B** Relative amounts of 2-alkanol ester homologs and isomers. **C** Correlation between ^13^C/^12^C ratios and plastidial carbon incorporation of different wax compositions. ^13^C/^12^C ratios are reported in standard delta notation as δ^13^C = (^13^C/^12^C sample—^13^C/^12^C standard)/^13^C/^12^C standard as permil (‰), where the standard is Vienna Pee Dee Belemnite. The δ^13^C values of compounds (carbon numbers given next to each data point) with known biosynthesis pathways were plotted as a function of their plastidial carbon percentages, assuming elongation in the plastids up to C_16_ and further elongation in the ER to the final chain lengths. Alkanes are formed by assembly of C_16_ chains in plastids and further carbon addition in the ER^2^, so the C_25_, C_27_, C_29_, and C_31_ alkanes contain 64%, 59.3%, 55.2%, and 51.6% plastidial carbon, respectively. Similarly, the 1-alkanol and 2-alkanol esters consist of alkyl and acyl moieties each incorporating a plastidial C_16_ precursor, and % of plastidial carbon can be calculated accordingly. The Ordinary Least Squares regression for the alkanes and esters is shown as solid line (*r*^2^ = 0.60), with 95% confidence intervals as dashed lines. Values for C_31_ β-diketone are plotted for the elongation hypothesis (Fig. 1) assuming 51.6% plastidial carbon (left) or the head-to-head condensation hypothesis predicting 100% plastidial carbon (right). Data are presented as means ± standard deviations of three biological replicates for (**A**, **B**) and three analytical replicates for (**C**). Source data are provided as a Source Data file.

GC-MS quantification showed that the spike wax β-diketones comprised 96.4% of C_31_ 14,16-diketone, along with 0.4% C_29_ 12,14-diketone, 0.3% C_29_ 14,16-diketone and 2.6% C_33_ 16,18-diketone (Fig. 2A and Supplementary Fig. S1). The C_30_ 14,16-diketone, C_32_ 14,16-diketone, and C_32_ 15,17-diketone made up 0.1%, 0.2%, and 0.1% of the fraction, respectively. The corresponding TLC fraction of barley flag leaf sheath wax had a very similar β-diketone composition (Supplementary Table S1). Overall, the barely β-diketones comprised predominantly C_16_ and C_18_ ketoacyl units (Fig. 1B, m = 11 or 13), confirming C_16_ 3-ketoacid and C_18_ 3-ketoacid as most likely pathway intermediates.

To further assess the chain length distributions of key intermediates for β-diketone formation, we analyzed the 2-alkanol esters known to be pathway side- products. In a TLC fraction of barley spike wax (R_f_ 0.73) both 1-alkanol esters and a series of 2-alkanol esters were identified. Among the 2-alkanol esters, the C_33_, C_35_, C_36_, and C_37_ homologs accounted for 8.3%, 58.5%, 3.0%, and 30.3%, respectively (Fig. 2B and Supplementary Table S2). Each of the four ester homologs comprised C_15_ 2-alkanol, amounting to 88%, 96%, 100%, and 59%, respectively (Fig. 2B). Across all homologs, the esters contained 84% C_15_ 2-alkanol, along with only 1% C_13_ and 14% C_17_ 2-alkanols. Similar 2-alkanol ester homolog distributions were observed in barley flag leaf sheath wax (Supplementary Table S3). Based on the general understanding that 2-alkanols are derived from corresponding 3-ketoacids one carbon longer^19,24^, the high concentration of C_15_ 2-alkanol esters suggests a precursor pool dominated by C_16_ 3-ketoacid rather than C_18_ 3-ketoacid.

### Mutant wax and carbon isotope analyses test elongation of the 3-ketoacid intermediate

To test whether β-diketone formation requires ER-localized VLC acyl elongation, we analyzed the wax of flag leaf sheaths of the *emr1* mutant lacking the core enzyme of the FAE complex responsible for wax precursor elongation, β-ketoacyl-CoA synthase 6 (KCS6/CER6). The mutant wax mixture showed a distinct elongation phenotype, with chain length distributions shifted to shorter homologs in comparison with the corresponding wild type (*H. vulgare* cv. Ingrid). All compound classes comprising chain lengths longer than C_24_ in the wild type (alkanes, 1-alkanols, 1-alkanol esters, and alkylguaiacols^34^) had profiles shifted towards shorter homologs in the mutant (Supplementary Fig. S4), similar to previous reports on the leaf wax of these lines^35^. However, the C_31_ β-diketones and hydroxy-β-diketones dominating the flag leaf sheath wax were found in similar concentrations in both lines. Formation of their carbon backbones is thus independent of the major wax ER-elongation enzyme, and our findings do not support elongation of a ketoacid precursor towards β-diketones.

Plant lipids have characteristic ^13^C depletion patterns known to encode information on growth conditions as well as biosynthetic mechanisms^36^. In particular, previous studies revealed differential isotope incorporation into wax compound classes and chain lengths in various biosynthesis steps^37–39^. Therefore, we aimed to analyze the isotope composition of β-diketones by GC coupled with isotope ratio mass spectrometry (IRMS), and to compare it with the isotope compositions of wax constituents formed along known biosynthesis pathways. The latter (alkanes and esters) had δ^13^C values positively correlated with the amounts of carbon incorporated during plastidial fatty acid synthesis (*r*^2^ = 0.60) (Fig. 2C). The β-diketones (>95% C_31_ 14,16-diketone) had δ^13^C −35.3 ± 0.3‰, a value fitting the trend of all other compound classes only if assuming 100% plastidial carbon content in the β-diketone. Thus, our isotope analysis further corroborates that β-diketone formation does not involve elongation of 3-ketoacid intermediates by ER-residing FAE enzymes (c.f. Fig. 1 and Supplementary Fig. S1).

### Thioesterase activity and chain length preference of barley DMH (HvDMH)

**Fig. 3:**
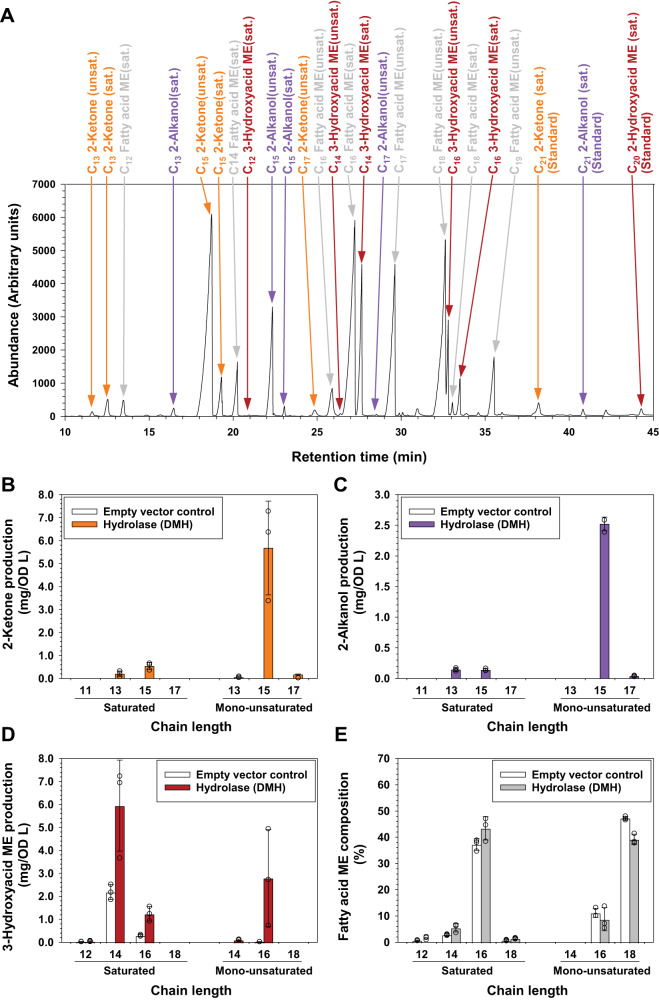
Characterization of the barley diketone metabolism hydrolase (HvDMH) producing the key intermediates on the β-diketone synthesis pathway. **A** Total ion chromatogram of lipids from *E. coli* expressing *HvDMH*, after transesterification into methyl esters (MEs) and conversion of hydroxyl groups into trimethylsilyl (TMS) ethers. Homolog series of saturated (sat.) and monounsaturated (unsat.) 2-ketones (orange), 2-alkanols (purple), 3-hydroxyacid MEs (red) and fatty acid MEs (gray) were detected. **B** Amounts of 2-ketones in *E. coli* expressing *HvDMH* (quantified against internal standard C_21_ 2-ketone). **C** Amounts of 2-alkanols in *E. coli* expressing *HvDMH* (quantified against internal standard C_21_ 2-alkanol). **D** Amounts of 3-hydroxyacid MEs in *E. coli* expressing *HvDMH* (quantified against internal standard C_20_ 2-hydroxyacid ME and normalized to C_16_ 3-hydroxyacid ME). **E** Amounts of fatty acid MEs in *E. coli* expressing *HvDMH*. Data are presented as means ± standard deviations of three biological replicates. Source data are provided as a Source Data file.

To further establish the chain length distribution of the β-diketone synthesis intermediates, we analyzed the product profiles of the barley DMH enzyme (HvDMH) thought to catalyze the first reaction of the pathway. GC-MS analyses of *E. coli* expressing *HvDMH* identified saturated and monounsaturated C_13_–C_17_ 2-ketones and 2-alkanols, along with C_12_–C_16_ 3-hydroxy fatty acids (Fig. 3A and Supplementary Fig. S5). The 2-alkanol and 2-ketone series were dominated by respective unsaturated C_15_ as well as saturated C_13_ and C_15_ compounds (Fig. 3B, C). Further MS analysis of dimethyldisulfide (DMDS) adducts identified the unsaturated 2-ketones as isomers with double bonds mainly at the ω−7 position (Supplementary Fig. S6). The series of 3-hydroxyacids comprised predominantly saturated C_14_ and C_16_ as well as unsaturated C_16_ compounds (Fig. 3D). None of the 2-ketones and 2-alkanols could be detected in corresponding empty-vector controls, while the concentrations of all 3-hydroxyacids were increased ~twofold from controls to *HvDMH*-expressing lines. As 2-ketones and 2-alkanols are likely formed by decarboxylation of respective 3-ketoacids and 3-hydroxyacids one carbon longer, our results imply that HvDMH generated predominantly C_16_ 3-ketoacid and 3-hydroxyacid in *E. coli*. These HvDMH chain length preferences matched our previous results showing that the wax β-diketones and 2-alkanol esters originated mainly from C_16_ ketoacyls.

To compare the chain length profiles of 3-keto-functionalized products with those of the available *E. coli* acyl pools, the latter were assessed by fatty acid methyl ester (FAME) analysis. The total lipids of the *HvDMH* expressors and empty-vector control lines had similar acyl profiles, with saturated C_12_–C_18_ fatty acyls peaking at C_16_ and corresponding unsaturated acyls peaking at C_18_ (Fig. 3E).

**Fig. 4:**
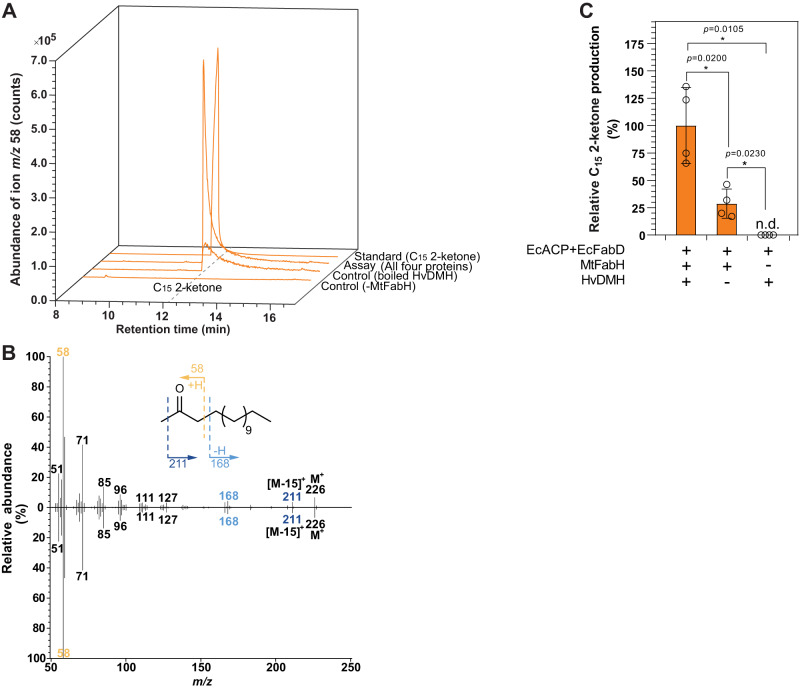
In vitro characterization of barley diketone metabolism hydrolase (HvDMH). **A** Gas chromatograms of the characteristic ion *m/z* 58 of 2-ketones. Trace of the standard is compared with that of extracted assay product. Barley HvDMH enzyme was incubated with *E. coli* acyl carrier protein (EcACP), *E. coli* malonyl-CoA:ACP transferase (EcFabD), and *Mycobacterium tuberculosis* 3-ketoacyl-ACP synthase (MtFabH), and with C_14_ fatty acyl-CoA and malonyl-CoA substrates. An identical assay with boiled HvDMH protein served as control for the hydrolase activity, and an assay lacking the MtFabH enzyme tested for background levels of 3-ketoacyl-ACP and its decarboxylation product C_15_ 2-ketone. **B** Mass spectra and fragmentation pattern of the C_15_ 2-ketone peaks of the HvDMH assay in (**A**, upper panel) and standard (lower panel). **C** Quantification of C_15_ 2-ketone in HvDMH assays shown in (**A**). Product amounts are normalized against the assay containing active HvDMH and MtFabH. Data are presented as means ± standard deviations of four biological replicates. Asterisks indicate significant differences between samples, as calculated by two-tailed Student’s *t* test; *P  <  0.05. Source data are provided as a Source Data file.

To corroborate the 3-ketoacyl thioesterase activity of HvDMH, we performed in vitro assays similar to those reported for characterization of tomato methylketone synthase^40,41^. In these coupled assays, purified *E. coli* acyl-carrier-protein (EcACP), *E. coli* malonyl-CoA:ACP transferase (EcFabD) and *Mycobacterium tuberculosis* 3-ketoacyl-ACP synthase (MtFabH) were used to convert malonyl-CoA substrate into malonyl-ACP and, together with C_14_ acyl-CoA as second substrate, further into C_16_ 3-ketoacyl-ACP. After incubation of this assay mixture with HvDMH, lipids were extracted and derivatized using elevated temperatures to decarboxylate the thioesterase product, sensitive 3-ketoacid, into corresponding 2-ketones for reliable quantification. In the assay product mixture, C_15_ 2-ketone was identified based on GC retention time (Fig. 4A) and MS fragmentation patterns (Fig. 4B) identical to those of an authentic standard. Control in vitro assays with boiled HvDMH protein yielded small amounts of C_15_ 2-ketone (Fig. 4C), while further control assays lacking MtFabH contained no detectable 2-ketone. Taken together, these assays thus defined a background of C_15_ 2-ketone formed by hydrolysis of C_16_ 3-ketoayl-ACP and decarboxylation of the resulting free 3-ketoacid, independent of HvDMH activity. Assays with the intact HvDMH enzyme contained significantly more C_15_ 2-ketone than the boiled-enzyme controls, thus showing the thioesterase activity of the enzyme (Fig. 4C). Overall, our assays demonstrated that HvDMH has in vitro thioesterase activity catalyzing the hydrolysis of 3-ketoacyl-ACP into free 3-ketoacid intermediates of β-diketone biosynthesis.

### Biochemical function of the barley DMP (HvDMP)

**Fig. 5:**
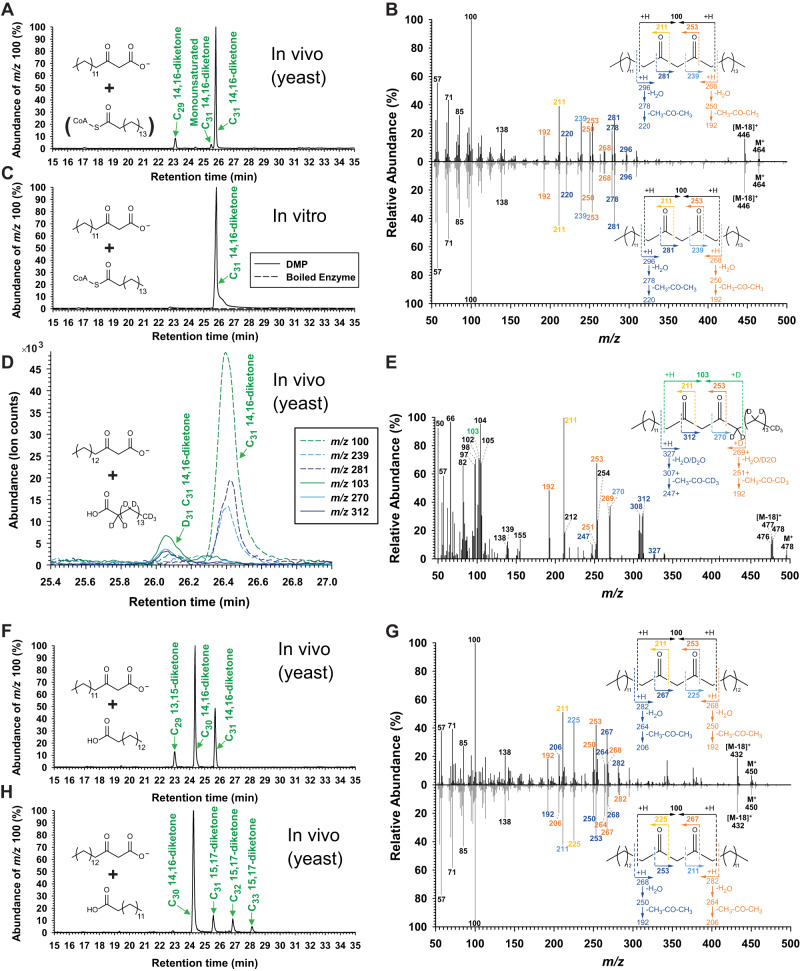
Characterization of the barley diketone metabolism polyketide synthase (HvDMP) catalyzing the condensation of 3-ketoacids and acyl-CoAs into β-diketones. GC-MS analysis of in vivo and in vitro assays feeding acyl-CoA and/or ketoacid substrates to HvDMP. Chromatograms of fragment *m/z* 100 selectively reporting β-diketones are shown on the left and mass spectra of key products on the right. **A** Chromatogram of lipids from yeast expressing *HvDMP* and supplemented with C_16_ 3-ketoacid (*n* = 11, 13). **B** Mass spectra of C_31_ 14,16-diketone peaks in (**A**, upper panel) and (**C**, lower panel). **C** Chromatogram of lipids from HvDMP in vitro assay with C_16_ 3-ketoacid and C_16_ acyl-CoA. Boiled recombinant enzyme incubated with C_16_ 3-ketoacid and C_16_ acyl-CoA served as control. **D** Chromatograms of characteristic fragments of lipids from yeast expressing *HvDMP* and supplemented with C_16_ 3-ketoacid and per-deuterated fatty acid C_15_D_31_COOH, selectively reporting undeuterated (dashed lines) and deuterated β-diketones (solid lines). **E** Mass spectrum of the D_31_-labeled C_31_ 14,16-diketone in (**D**), showing α-fragment, McLafferty rearrangement and water/acetone loss ions diagnostic for the C_14_ acyl moiety of C_31_ 14,16-diketone. Analogous fragments of the other hydrocarbon tail were shifted by 31 amu relative to the unlabeled C_31_ 14,16-diketone, as were the molecular ion and the water loss ion [M-18]^+^. A cluster of ions around *m/z* 103 is consistent with double-McLafferty rearrangement on both sides of the β-diketo group, a double-deuterated α-methylene and γ-deuterium transfer. Together, the GC and MS characteristics unambiguously identified this compound as C_31_ 14,16-diketone with C_13_H_27_ and C_15_D_31_ hydrocarbon tails. **F** Chromatogram of lipids from yeast expressing *HvDMP* and supplemented with even-numbered 3-ketoacid (C_16_) and odd-numbered fatty acid (C_15_). **G** Mass spectra of C_30_ 14,16-diketone in (**F**, upper panel) and (**H**, lower panel). **H** Chromatogram of lipids from yeast expressing *HvDMP* and supplemented with odd-numbered 3-ketoacid (C_17_) and even-numbered fatty acid (C_14_). Arabidopsis *LACS1* was expressed in all the yeast assays to enhance exogenous substrate uptake^61^.

Based on all the evidence suggesting C_16_ 3-ketoacid as central intermediate of the β-diketone pathway, our next goal was to test its role as a substrate of the ensuing step and, thus, to establish the biochemical activity of HvDMP. However, the expression of HvDMH in yeast (*Saccharomyces cerevisiae*) did not lead to the production of the ketoacid, so the compound had to be supplemented exogenously. When C_16_ 3-ketoacid was fed to yeast expressing *HvDMP*, three compounds were detected which were not present in controls expressing empty vector or lacking substrate. Based on their GC-MS characteristics, the major product was identified as C_31_ 14,16-diketone, along with C_29_ 14,16-diketone and a monounsaturated C_31_ 14,16-diketone (Fig. 5A, B). Thus, HvDMP proved active in yeast, likely condensing C_16_ 3-ketoacid directly with C_16_ fatty acyl to form C_31_ 14,16-diketone.

To corroborate the biochemical activity of HvDMP, the recombinant enzyme was assayed in vitro with various substrate combinations. Incubation with C_16_ ketoacid and C_16_ fatty acyl-CoA gave a single product, identified as C_31_ 14,16-diketone (Fig. 5B, C). In contrast, corresponding assays of HvDMP with varying molar ratios of C_16_ ketoacid and malonyl-CoA yielded no detectable β-diketone or triketide/tetraketide products. Incubation of the enzyme with C_16_ fatty acyl-CoA and malonyl-CoA in diverse concentrations also did not give detectable products. Together, these findings confirm that HvDMP uses a ketoacid instead of malonyl-CoA extenders for condensation with a fatty acyl-CoA.

To test the HvDMP-catalyzed condensation of the two substrates further, we fed per-deuterated C_16_ fatty acid together with C_16_ ketoacid to yeast expressing the enzyme. The resulting lipids contained unlabeled C_31_ 14,16-diketone together with a novel compound with shorter GC retention time (Fig. 5D). The MS characteristics (Fig. 5E and Supplementary Fig. S7) unambiguously identified this compound as C_31_ 14,16-diketone with one normal (C_13_H_27_) and one per-deuterated hydrocarbon tail (C_15_D_31_) ﻿originating from the labeled fatty acid. The formation and exact structure of this product indicated the direct incorporation of the entire acyl moiety into the β-diketone product and further confirmed that HvDMP catalyzed its condensation with ketoacid also in vivo.

To test whether HvDMP condensed entire ketoacid and fatty acyl-CoA units, we assayed the enzyme with substrates having unusual odd-numbered acyl chains. In one experiment, feeding C_15_ fatty acid and C_16_ ketoacid to yeast expressing *HvDMP* yielded three compounds absent from empty-vector controls, the major compound being C_30_ 14,16-diketone and the two minor ones C_29_ 14,16-diketone and C_31_ 14,16-diketone (Fig. 5F, G). In a similar assay, C_17_ ketoacid and C_14_ fatty acid were fed to *HvDMP*-expressing yeast, resulting in the formation of C_30_ 14,16-diketone as a major product together with C_31_ 15,17-diketone, C_32_ 16,18-diketone and C_33_ 15,17-diketone (Fig. 5G, H). The finding that each of these assays yielded several products sharing one acyl moiety, taken together with the structure of respective major β-diketone products, further confirmed that HvDMP catalyzed the direct condensation of fatty acyl-CoA and ketoacid substrates.

### Chain length preference of HvDMP for fatty acyl-CoA and 3-ketoacid substrates

**Fig. 6:**
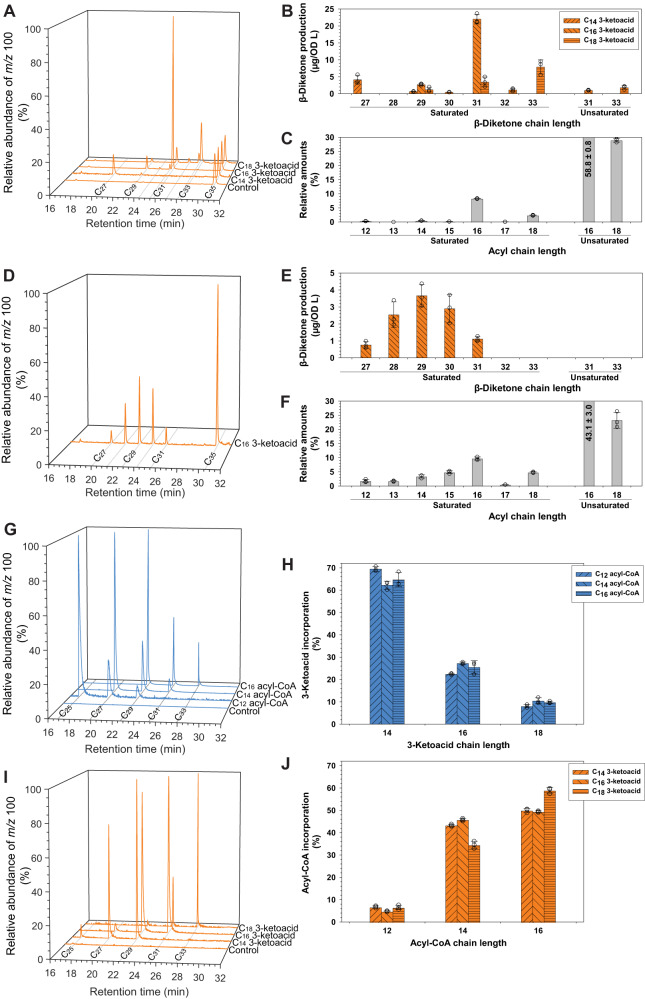
Assessment of HvDMP substrate preferences. GC-MS analysis of in vivo and in vitro assays feeding several acyl-CoA and/or ketoacid substrates to HvDMP. Chromatograms of fragments *m/z* 100 selectively reporting β-diketones are shown on the left and corresponding β-diketone product profile(s) on the right. **A** Selected-ion chromatograms of lipids from yeast expressing *HvDMP* and supplemented with C_14_, C_16_, or C_18_ 3-ketoacid, or only buffer. **B** β-diketone profiles produced in (**A**) (quantified against internal standard C_35_ 14,16-diketone). **C** Relative composition of fatty acyl pools in yeast expressing *HvDMP* (quantified after transmethylation of total lipid extracts of yeast not supplemented with ketoacid). **D** Selected-ion chromatogram of lipids from yeast expressing *HvDMP* and supplemented with C_16_ 3-ketoacid alongside C_12_–C_15_ fatty acids (to adjust the fatty acyl profile). **E** β-diketone profiles produced in (**D**) (quantified against internal standard C_35_ 14,16-diketone). **F** Relative composition of fatty acyl pools in yeast expressing *HvDMP* (quantified after transmethylation of total lipid extracts of yeast supplemented with C_12_ to C_15_ fatty acids). **G** Selected-ion chromatograms of lipids from HvDMP in vitro assays combining equal molar concentration of C_14_–﻿C_18_ 3-ketoacids alongside C_12_, C_14_ or C_16_ fatty acyl-CoA. Boiled recombinant enzyme incubated with equimolar concentrations of C_14_–﻿C_18_ 3-ketoacids alongside C_16_ fatty acyl-CoA served as control. **H** Proportions of 3-ketoacids incorporated with each fatty acyl-CoA co-substrate into the β-diketones shown in (**G**). **I** Selected-ion chromatograms of lipids from HvDMP in vitro assays combining equal molar concentration of C_12_–﻿C_16_ fatty acyl-CoAs with C_14_, C_16_, or C_18_ 3-ketoacid. Boiled recombinant enzyme incubated with equimolar concentrations C_12_–﻿C_16_ fatty acyl-CoAs and C_16_ 3-ketoacid served as control. **J** Proportions of fatty acyl CoAs incorporated with each 3-ketoacid co-substrate into the β-diketones shown in (**I**). Arabidopsis *LACS1* was expressed in all the yeast assays to enhance exogenous substrate uptake^61^. Data are presented as means ± standard deviations of three biological replicates. Source data are provided as a Source Data file.

Four experiments were performed to test the chain length preferences of HvDMP. In a first set of assays, we supplemented yeast expressing *HvDMP* with equal molar amounts of C_14_, C_16_, or C_18_ ketoacids and quantified the resulting β-diketone products. Feeding of C_14_ 3-ketoacid led to a β-diketone mixture comprising mainly C_27_ 12,14-diketone, whereas C_16_ 3-ketoacid feeding afforded mainly C_31_ 14,16-diketone, and C_18_ 3-ketoacid mainly C_33_ 16,18-diketone (Fig. 6A, B). Thus, the HvDMP preferences for acyl-CoA substrates depended on the 3-ketoacid present, resulting in condensation of C_14_ 3-ketoacid mainly with C_14_ acyl-CoA, C_16_ 3-ketoacid with C_16_ acyl-CoA, and C_18_ 3-ketoacid with C_14_ and C_16_ acyl-CoAs.

To gauge the effect of fatty acyl chain lengths available in the in vivo assays, we quantified the fatty acyls in the total lipid mixture of the yeast lines (as FAMEs). The acyl pools of control yeast (without substrate feeding) comprised mainly monounsaturated C_16_ and C_18_ chains (together 88%), along with saturated C_12_–C_18_ acyls dominated by C_16_ (8%) and C_18_ (3%) (Fig. 6C). Compared with this profile of available substrates, HvDMP greatly preferred saturated over unsaturated acyl substrates in our assays, and to some degree C_14_ acyls over the more abundant C_16_ and C_18_ homologs (Supplementary Fig. S8A).

The preference of HvDMP for certain acyl-CoA chain lengths was assessed in a second in vivo experiment where we supplemented yeast expressing the enzyme with different fatty acid homologs. Preliminary tests showed that co-feeding of C_16_ 3-ketoacid with either C_11_ or C_17_ fatty acids yielded the same β-diketones as controls lacking exogenous acids. However, co-feeding of C_16_ 3-ketoacid with a mixture of C_12_–C_15_ fatty acids led to production of C_27_–C_31_ β-diketones (Fig. 6D). The products had a near-symmetric chain length distribution centered at C_29_, including substantial amounts of two even-numbered β-diketones, C_28_ and C_30_ (Fig. 6E). In contrast to the controls lacking exogenous fatty acids, unsaturated β-diketones could not be detected in this assay. Fatty acid profiling of the total yeast lipids confirmed substantially increased amounts of C_12_–C_15_ acyls in the complemented assays (Fig. 6F). Overall, HvDMP thus condensed C_16_ 3-ketoacid with fatty acyls ranging from C_12_ to C_16_, but preferring C_13_/C_14_ acyls (Supplementary Fig. S8B).

Finally, the interdependence between the HvDMP 3-ketoacid and acyl substrate chain length preferences was explored using in vitro assays with defined, limiting substrate concentrations. In one set of experiments, recombinant HvDMP was incubated with a mixture containing one molar equivalent each of C_14_, C_16_, and C_18_ ketoacids and 1.5 equivalents of a particular fatty acyl-CoA (C_12_, C_14_, or C_16_). In all three assays, over 60% of the β-diketone products were formed by incorporation of C_14_ 3-ketoacid substrate, accompanied by 20% of C_16_ 3-ketoacid condensation products and circa 10% of C_18_ ketoacid products (Fig. 6G, H). The HvDMP enzyme, thus, had a strong preference for C_14_ 3-ketoacid, irrespective of acyl-CoA co-substrate chain length.

In a final set of experiments, the acyl-CoA preference of HvDMP was tested in competition assays with limited amounts of 3-ketoacid co-substrates, where the enzyme was incubated with a mixture of C_12_, C_14_ and C_16_ fatty acyl-CoAs (1:1:1 molar ratio) and either C_14_, C_16_, or C_18_ 3-ketoacid (1.5 molar equivalents). In all three assays, C_16_ fatty acyl-CoA substrate was slightly preferred over C_14_ fatty acyl-CoA, and less than 7% of the β-diketones originated from C_12_ acyl-CoA (Fig. 6I, J). Overall, the in vitro assays thus showed that HvDMP preferentially condensed C_14_ 3-ketoacid with C_14_ or C_16_ fatty acyl-CoA to form C_27_ or C_29_ 14,16-diketone, respectively.

### Subcellular compartmentation of the β-diketone-forming enzymes

**Fig. 7:**
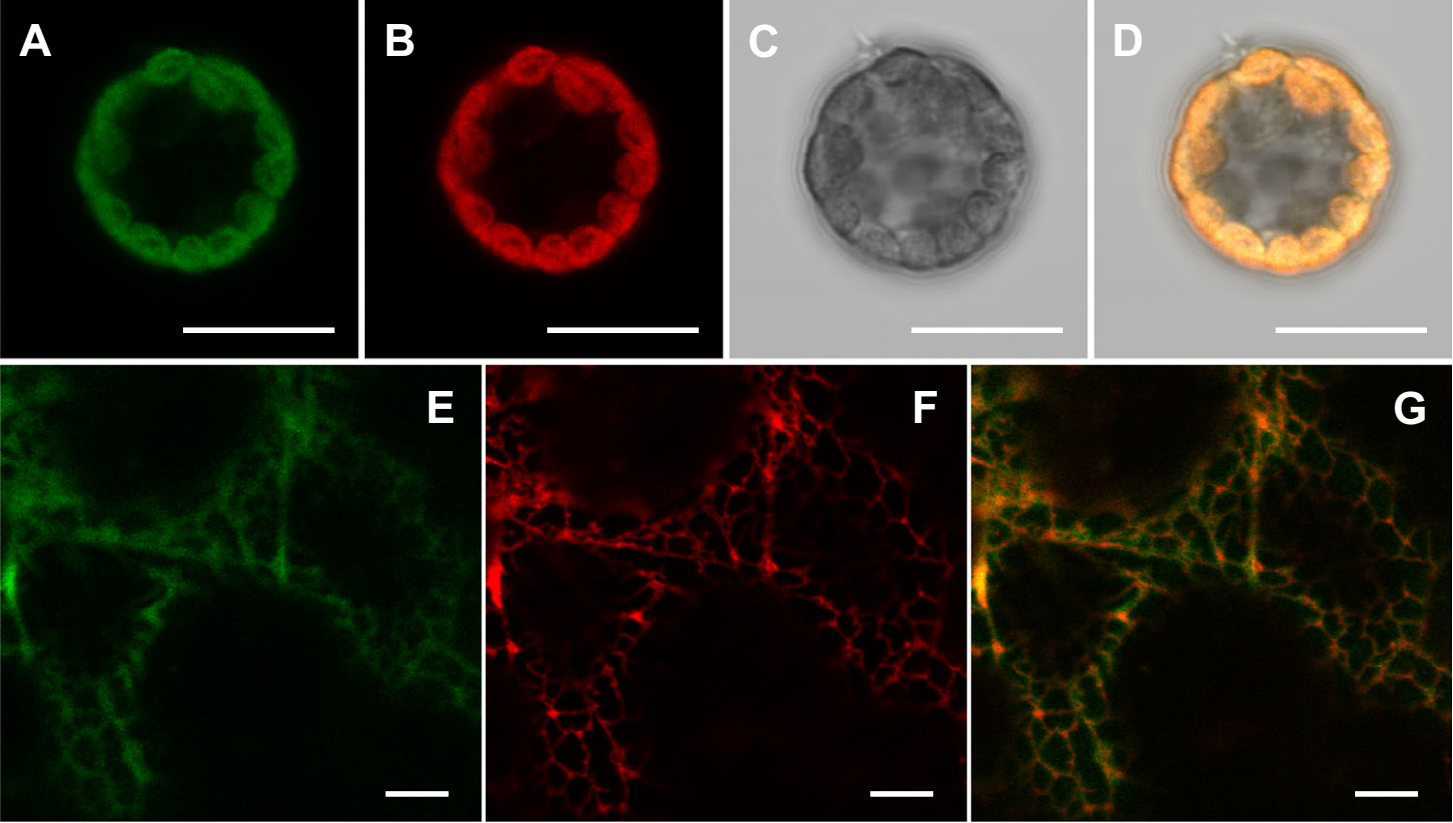
Subcellular localization of HvDMH and HvDMP. Confocal microscope images of barley protoplasts transiently expressing *Pro35S:HvDMH-GFP*, showing **A** HvDMH*-*GFP, **B** chloroplast autofluorescence, **C** bright field, and **D** the merged image of (**A**–**C**). Confocal microscope images of *N. benthamiana* leaves coexpressing, **E** *Pro35S:GFP-HvDMP*, and **F** the ER marker *Pro:35S:HDEL-RFP*, and **G** merged image of (**E**, **F**). Bars =  10 μm. Both experiments were independently repeated three times and 10–15 samples/regions were investigated each time with similar results.

Based on our biochemical results showing that only two enzymes, HvDMH and HvDMP, were required for β-diketone formation, we aimed to localize the biosynthetic pathway across the cellular compartments. For this, we first determined the subcellular localization of HvDMH by confocal microscopy of barley protoplasts transiently expressing green fluorescent protein (GFP) fusion constructs. The resulting GFP fluorescence signal matched the chlorophyll autofluorescence patterns within the cells (Fig. 7A–D), indicating that this enzyme resided in the chloroplast. In contrast, transient expression of GFP-HvDMP fusion proteins in tobacco leaves showed a reticulate pattern and colocalized with ER-specific marker HDEL-RFP (Fig. 7E–G), suggesting that the HvDMP protein resides in the ER.

## Discussion

The major findings of our experiments were that (i) C_16_ 3-ketoacid is the central intermediate en route to barley β-diketones, (ii) DMH generates fatty acid derivatives with functional groups on C-3, and (iii) DMP uses 3-ketoacids and fatty acyl-CoAs for head-to-head condensation into β-diketones. These results can now be integrated to describe the overall biochemistry of the β-diketone biosynthesis pathway.

### Characterization of the first pathway step

Initial information on the β-diketone-forming pathway came from our barley wax analyses. First, we confirmed previous reports that barley spike and flag leaf sheath waxes contained mainly C_15_ 2-alkanol esters^18,27^. Since wax 2-alkanols are thought to be derived from 3-ketoacids one carbon longer^24^, this finding suggests that the C_16_ 3-ketoacid is a major precursor available in planta. Secondly, we also confirmed earlier results on the composition of β-diketones with odd carbon numbers^8^ and further identified unbranched, even-numbered β-diketones. The latter are distinguished by one hydrocarbon tail that can only be formed by elongation from an initial C_3_ (rather than C_2_) moiety, and thus must have been present before functional group formation. In particular, our MS analyses showed that these β-diketones have functional groups on C-15/C-17 and C-17/C-19, implying that respective C_15_, C_17_, or C_19_ moieties must be synthesized prior to the diketo group. This left two scenarios, where the odd-numbered acyls may be either C_17/19_ 3-ketoacyl or C_15/17_ acyl intermediates (see Fig. 1B). Interestingly, the latter scenario implies head-to-head condensation with C_16_ 3-ketoacid and, thus, fits our previous finding that C_16_ 3-ketoacid is the major pathway intermediate. This also matches earlier reports that feeding C_15_ acyl substrate to barley tissue slices led to the formation of C_30_ 14,16-dione^8^, one of the even-numbered β-diketones identified here in barley wax.

Our chemical datasets were complemented by biochemical assays with HvDMH, the enzyme catalyzing the first committed step of the β-diketone pathway^19,20^. Experiments using *E. coli* heterologous expression confirmed the thioesterase activity of HvDMH, and the overall product profiles of HvDMH (in comparison with the acyl pools present) showed that the enzyme had a strong preference for C_14_ and C_16_ acyl substrates. The thioesterase activity of HVDMH was further corroborated using coupled in vitro assays with C_16_ 3-ketoacyl-ACP substrate. The biochemical assays, therefore, underscored the conclusion that C_16_ 3-ketoacid was the major intermediate incorporated into the various β-diketone homologs and isomers.

It is of note that HvDMH expression in *E. coli* yielded high amounts of unsaturated 2-ketones, 2-alkanols, and 3-hydroxyacids, showing that the enzyme does not discriminate between substrates with specific geometry of aliphatic tails including C = C double bonds. Our results suggest that the unsaturated 2-ketones are formed from ω−7 monounsaturated 3-ketoacids which are direct hydrolysis products of corresponding acyl-ACP intermediates of *E. coli* fatty acid synthesis^42^. However, plants typically produce much smaller proportions of unsaturated C_16_ acyls than *E. coli*^43^, so that HvDMH will likely encounter lower concentrations of this substrate in plant epidermal cells than in our bacterial assays. The details of our heterologous expression experiment can, therefore, not be directly extrapolated to infer the in planta activity of HvDMH.

In contrast to the HvDMH assays in *E. coli*, HvDMH upon expression in yeast failed to produce 3-ketoacyl intermediates or their derivatives under the tested conditions. It seems plausible that the plant enzyme, HvDMH, may successfully intercept intermediates only from type-II FAS complexes present in plants and *E. coli*^44^, but not the type-I FASs present in yeast^45^. However, it is also possible that the hydrolase requires specific ACP isoforms as substrates^46^ and therefore discriminates against yeast ACPs.

Altogether, our wax analyses and HvDMH assays firmly established several homologous 3-ketoacids as key intermediates of the β-diketone biosynthesis pathway, with C_16_ 3-ketoacid as the major substrate for ensuing reactions. However, our data thus far did not allow us to distinguish between the elongation and head-to-head condensation hypotheses, and to test these alternatives we investigated the second pathway reaction catalyzed by HvDMP.

### Characterization of the second pathway step

We initially sought to test the elongation hypothesis with chemical evidence. To this end, we first analyzed the waxes of the barley *emr1* mutant deficient in the condensing enzyme of the FAE complex, KCS6/CER6^35^. However, the mutant had β-diketone amounts and profiles similar to those of the wild type, suggesting that β-diketone biosynthesis must either involve an elongation machinery largely independent of that forming all other wax compounds or else may not rely on ER-based elongation mechanisms at all. This conclusion confirmed previous reports that β-diketone biosynthesis is not influenced by hydrocarbon elongation inhibitors^25^. Second, we gauged the portions of carbon atoms incorporated in the ER, the compartment known to harbor all VLC elongation enzymes^2^, against carbons incorporated in the plastids during initial fatty acyl assembly. Our results revealed a positive correlation of ^13^C isotope values with plastidial carbon proportions across all barley wax compound classes with known biosynthesis mechanisms, similar to trends reported before for some but not all wax mixtures of other species^39,47,48^. The ^13^C value of barley wax β-diketone very closely matched that predicted assuming that all its carbons are of plastidial origin and clearly differed from that predicted for ER elongation of a C_16_ precursor. The isotope evidence, thus, favored the head-to-head condensation hypothesis for the second step of the β-diketone biosynthesis pathway over the elongation hypothesis.

To test the head-to-head condensation hypothesis in detail, we assayed the activity of the key enzyme of the β-diketone pathway, HvDMP. Feeding of C_16_ 3-ketoacid to yeast expressing *HvDMP* led to the formation of C_31_ 14,16-diketone and, thus, showed overall condensation activity. Corresponding yeast assays supplemented by deuterium-labeled or odd-numbered fatty acids unambiguously showed that the entire chain of the acyl substrate was incorporated into the β-diketones. Similarly, feeding of characteristic 3-ketoacid and fatty acid further confirmed that both precursors were directly condensed into the product. These findings were finally corroborated by in vitro assays where HvDMP incorporated C_16_ 3-ketoacid and C_16_ fatty acyl-CoA directly into C_31_ 14,16-diketone. Together, our experiments thus characterized HvDMP as a condensing enzyme utilizing long-chain acyl-CoA starter and 3-ketoacid extender, in lieu of the aromatic acyl-CoA starters and malonyl-CoA extenders of most other plant PKSs^28,44,45^.

Only few non-canonical plant PKSs had previously been shown to catalyze reactions somewhat similar to HvDMP (Supplementary Fig. S9). For example, *Curcuma longa* CURSs condense a coumaroyl-CoA or feruloyl-CoA starter with a coumaroyldiketide or feruloyldiketide acid extender^32,49^, while the *Aquilaria sinensis* phenylethylchromone-forming polyketide synthase (PECPS) condenses benzoyl-CoA starters with aromatic extender^50^. In contrast, the alkylquinolone synthase (AQS) from *Tetradium ruticarpum* (formerly *Euodia ruticarpa*) combines a characteristic aromatic starter, *N*-methylanthraniloyl-CoA, with a medium-chain 3-ketoacid en route to alkaloid products^51^. Similar activities involving aromatic starters and aliphatic extenders were also shown, albeit only in vitro, for rice (*Oryza sativa*) curcuminoid synthase (CUS)^52,53^, multiple PKSs from *A. sinensis* and a *Huperzia serrata* PKS (HsPKS)^54–56^. Overall, these enzymes catalyze comparable condensation reactions not involving malonate but instead using extenders similar to their (mostly aromatic) starters. HvDMP also condenses two substrates that are similar to each other, however both are long-chain compounds very different from those of the other PKSs.

Interestingly, HvDMP shares 55% similarity with CUS, 42% with CURS, 42% with AQS, 37% with HsPKS3, 44% with PECPS and 44% with *Medicago sativa* CHS^32,50–52,55,57^, and sequence comparisons clearly identify a Cys-His-Asn catalytic triad in HvDMP essential for plant PKSs^31^. Accordingly, we propose a reaction mechanism analogous to other condensing enzymes^53^, proceeding via initial covalent binding of the starter acyl, entry of the ketoacid extender and decarboxylative Claisen condensation between both substrates (Supplementary Fig. S10). It will be interesting to determine the tertiary structure of HvDMP, for comparison of its active site cavity with those of plant PKSs using substrates with very different molecular geometry and polarity.

### Context of the β-diketone biosynthesis pathway

Finally, the structures of the β-diketones accumulating in barley waxes can be explained based on the biochemistry of the two key enzymes and a comparison of the substrate preferences of both pathway steps. HvDMP prefers C_14_ 3-ketoacid substrate but also readily accepts the C_16_ 3-ketoacid mainly produced by HvDMH. Similarly, HvDMP has high activity on saturated C_14_ fatty acyl co-substrates, but also on the C_16_ fatty acyls predominating its substrate pool in planta^58,59^. Both pathway enzymes, thus, have sufficient activity on their respective C_16_ substrates to make the preponderance of this chain length in the acyl pool translate into its strong dominance in the final β-diketone homolog and isomer composition. This explains why the one β-diketone generated from C_16_ acyl and C_16_ 3-ketoacyl moieties, C_31_ 14,16-diketone, makes up more than 96% of the product mixture in barley.

The 3-ketoacid generated by HvDMH in plastids must be exported to the ER, the subcellular compartment of the HvDMP, and the 3-ketoacids must be chemically stable enough to survive this transport process (likely occurring by diffusion of the lipophilic compound in respective bilayer membranes). In this context, it is of note that the 3-ketoacids proved fairly stable at room temperature in our in vivo and in vitro assays, but were prone to decarboxylation at elevated temperatures as previously reported^19^. The second substrate required by HvDMP, the long-chain fatty acyl, is also formed in the plastids and exported to the ER, with CoA thioesterification occurring in the process^2,60^. Assembly of β-diketones then occurs by HvDMP, 3-ketoacid and acyl-CoA all coming together in the ER, where also all other wax compounds are synthesized^2^. However, the formation of the other wax constituents there proceeds in small increments gradually increasing the lipophilicity of the compounds, whereas β-diketone synthesis is accomplished in a single step going directly from long-chain precursors to a highly lipophilic VLC hydrocarbon structure.

Taking all our findings together, we propose a model for the biosynthesis of β-diketones and their associated wax compounds, where (i) HvDMH acts as a thioesterase that intercepts 3-ketoacyl-ACPs from type-II FAS complexes to produce 3-ketoacids in plastids of epidermis cells; (ii) the 3-ketoacids are then exported to the ER, likely through bilayer membrane contact sites and/or hemifusions; (iii) fatty acyl-CoAs derived from plastidial fatty acid de novo synthesis are also imported into the ER; (iv) in the ER, the DMP enzyme catalyzes a decarboxylative head-to-head condensation of both substrates to form β-diketones; (v) most of the β-diketones are exported directly to the plant surface, while (vi) some are, likely still in the ER, first hydroxylated to hydroxy-β-diketones by HvDMC and then exported (Fig. 1A). In a side reaction, part of the 3-ketoacid intermediates may be decarboxylated either spontaneously or by a decarboxylase into 2-ketones, which are reduced to 2-alkanols and esterified with fatty acyl-CoAs to form 2-alkanol esters. It cannot be ruled out that the 2-alkanols are formed by spontaneous or enzymatic decarboxylation of 3-hydroxyacids (originating from HvDMH-catalyzed hydrolysis of 3-hydroxyacyl-ACPs).

Since the formation of the β-diketones and their derivatives recruits substrates directly from fatty acid synthesis, it must compete with co-occurring processes of primary lipid metabolism. Therefore, β-diketone biosynthesis must be tightly regulated, especially during the expansion of epidermal cells in the course of organ growth. In this context, it is of particular interest that the β-diketone biosynthesis genes are clustered in the barley and wheat genomes^19,20,23^, and their expression is under tight control by inhibitory elements in wheat^23^. It will be interesting to investigate the regulatory mechanisms in the context of primary lipid metabolism. It will also be interesting to compare mechanisms of β-diketone formation between barley and diverse other species known to also accumulate these compounds in their surface waxes.

In conclusion, the evidence provided here characterizes HvDMP as a PKS condensing non-aromatic acyl-CoA starters with unusual ketoacid extenders to directly form the β-diketones accumulating in barley leaf and spike surface waxes. The unique ketoacid substrates are generated by a specific thioesterase, HvDMH, intercepting intermediates of fatty acid biosynthesis in the plastid. The homolog and isomer distribution of the barley wax β-diketones are fully explained by the combined chain length preferences of both enzymes and the available fatty acyl pool. The biosynthesis pathway to β-diketones is, thus, very distinct from pathways leading to other wax compounds found ubiquitously in the wax mixtures of plants. Only two enzymes define the entire pathway to β-diketones and their derivatives, in stark contrast to the previously assumed multi-step process relying on incremental elongation of the β-diketone carbon backbone. These insights into the biosynthesis of major components of Poaceae cuticles will enable further research towards breeding of cereal crop lines with improved drought tolerance.

## Methods

### Chemicals

All chemicals were obtained from Sigma-Aldrich, unless specified otherwise below.

### Plant materials and growth conditions

*H. vulgare* cv. Morex (NGB23015) seeds were obtained from the Nordic Genetic Resource Center (Alnarp, Sweden), seeds of *H. vulgare* cv. Ingrid and *emr1* were kindly provided by Dr. U. Schaffrath (Technische Hochschule Aachen, Germany). Seeds were germinated on moist filter paper in Petri dishes at room temperature, then transferred to soil (Sunshine Mix 5, Sun Gro) in 10-l pots. Plants were grown in a growth chamber under 16-h light/21 °C, 8-h dark/19 °C cycles. For barley protoplast preparation, barley cv. Morex seeds were germinated on moist filter paper in Petri dishes, and plantlets were transferred to soil in small pots covered with plastic domes and kept in the growth chamber for 10 days under the conditions described above. Tobacco (*Nicotiana benthamiana*) plants grown under the same conditions for 1 month were used for leaf transient expression.

### RNA isolation, reverse transcription, plasmid construction, and transformation

Flag leaf sheaths and spikes of *H. vulgare* cv. Morex were excised and immediately frozen in liquid nitrogen, total RNA was extracted using PureLink RNA mini kit (Invitrogen), and genomic DNA was removed by on-column DNA digestion with PureLink DNase (Invitrogen) following the manufacturer’s protocol. In total, 1 µg of resulting total RNA and Oligo(dT)_20_ primer (Invitrogen) was used to synthesize first-strand complementary DNA (cDNA) with SuperScript III Reverse Transcriptase (Invitrogen). The resulting cDNA served as a template for gene cloning.

*E. coli* expression vectors pET28-HvDMH, pET28-HvDMP and pET28-TEVH were kindly provided by Dr. A. Aharoni (Weizmann Inst., Rehovot, Israel). The expression cassette contains an N-terminal 6xHis-tag followed by a TEV cleavage site and the open reading frame (ORF) of the target gene. To construct *E. coli* expression vectors pET28a-EcACP, pET28a-EcFabD and pET28a-MtFabH, EcACP was amplified from *E. coli* (DH 5α) with Phusion polymerase (NEB) using primers EcACP-NdeI-F/EcACP-BamHI-R (Supplementary Table S4). The EcFabD and MtFabH sequences were synthesized by Twist Bioscience and further amplified using primers EcFabD-NdeI-F/EcFabD-BamHI-R and MtFabH-NdeI-F/MtFabH-BamHI-R, respectively. The PCR products were purified and inserted into pET28a vector and in frame with N-terminal 6xHis-tag followed by a thrombin cleavage site. *E. coli* transformed with target vector were screened on lysogeny broth (LB) plates containing 50 µg/ml kanamycin, and verified by colony PCR, restriction digestions, and Sanger sequencing. The confirmed expression vectors were transformed into *E. coli* BL21 (DE3) competent cells (Invitrogen) according to the manufacturer’s protocol, and the presence of the gene insert was verified by colony PCR. Three to four independent transformants of each *E. coli* line were selected for further in vivo assay or protein purification.

To construct plant expression vectors, the HvDMH and HvDMP coding regions were amplified from *H. vulgare* cv. Morex flag leaf sheath cDNA with primers HvDMH-F/HvDMH-R and HvDMP-F/HvDMP-R, respectively (Supplementary Table S4). PCR products were ligated to Gateway entry vector pCR8/GW/TOPO (Invitrogen) according to the manufacturer’s protocol, and the presence of the target sequences was verified by colony PCR and Sanger sequencing. LR reactions were performed with LR Clonase II enzyme mix (Invitrogen) to transfer target genes from the entry vector to the destination vector for in-frame C-terminal or N-terminal fusion with GFP. Positive transformants were identified by colony PCR and Sanger sequencing. The resulting constructs were transformed into *Agrobacterium tumefaciens* GV3101 competent cells by electroporation, screened on LB plates containing proper antibiotics, and verified by colony PCR. Three verified transformants were cultured for tobacco transient expression.

To construct yeast expression vectors, the coding sequence of long-chain acyl-CoA synthetase 1 (LACS1) that enhances yeast lipid uptake^61^ was amplified from Arabidopsis stem cDNA using primers LACS1-BamHI-F and LACS1-SalI-R and inserted into the pESC-Trp yeast expression vector in-frame with C-terminal MYC epitope tag, for expression under control of the galactose-inducible promoter GAL1, resulting in construct pESC-Trp-GAL1::LACS1-MYC. HvDMP was subcloned from pGWB6-35Spro::GFP-HvDMP using primers HvDMP-EcoRI-F and HvDMP-SpeI-R and spliced into pESC-Trp-GAL1::LACS1-MYC vector, C-terminally in-frame with FLAG epitope tag, resulting in construct pESC-Trp-GAL1::LACS1-MYC-GAL10::HvDMP-FLAG. *E. coli* transformed with either vector were screened on LB plates containing 100 µg/ml ampicillin, and verified by colony PCR, restriction digestions and Sanger sequencing. The resulting expression vectors were transformed into yeast strain INVSc1 (*MATα/MATa his3Δ1/ his3Δ1 leu2/leu2 trp1-289/trp1-289 ura3-52/ura3-52*) as described^62^, and the resulting transformants were screened on yeast minimal medium plates lacking the appropriate amino acids. The transformed yeast colonies were verified by colony PCR. Three to four verified yeast transformants were selected for further in vivo assays.

### Chemical synthesis of standards and substrates

#### C_31_ 14,16-diketone (hentriacontane-14,16-dione)

In all, 1 g of hexadecanol was stirred at room temperature with 2.5 g of Dess-Martin reagent periodinane in 30 ml CH_2_Cl_2_. Upon completion of the oxidation (monitored by TLC), the reagent was quenched with aqueous Na_2_S_2_O_3_ solution. The mixture was extracted with CH_2_Cl_2_, and the combined organic fractions were washed with aqueous NaH_2_CO_3_ solution, dried with Na_2_SO_4_ and concentrated in vacuo. The product, hexadecanal, was purified by column chromatography (CHCl_3_) and verified by GC-MS analysis.

In all, 0.7 g 2-pentadecanone was stirred at 0 °C with 0.1 M lithium diisopropylamide in 11.5 ml tetrahydrofuran (THF) for 10 min, then 2 ml THF containing 0.8 g hexadecanal were added drop-wise, and after 1 h the reaction was quenched with 5 ml 20% H_2_SO_4_. The mixture was extracted with CH_2_Cl_2_, and the combined organic solutions were washed, dried and concentrated described as above. The product, 16-hydroxyhentriacontan-14-one, was purified by column chromatography (1:1 hexane:CHCl_3_) and verified by GC-MS analysis.

In all, 0.5 ml 1 M oxalyl chloride in CH_2_Cl_2_ was added drop-wise to 150 µl dimethyl sulfoxide (DMSO) in 2 ml CH_2_Cl_2_ under stirring at −78 °C. After gas evolution had ceased, 0.2 g 16-hydroxyhentriacontan-14-one in 3 ml CH_2_Cl_2_ were added drop-wise, the reaction mixture was allowed to warm to room temperature, and then 0.3 ml Et_3_N was added. The resulting solid was re-dissolved by adding 5 ml water, and organic products were extracted with CH_2_Cl_2_. The combined organic phases were washed first with diluted HCl and then with water, dried with Na_2_SO_4_, and concentrated. The resulting product, hentriacontane-14,16-dione, was purified by column chromatography (3:1 hexane: CHCl_3_) and verified by MS analysis.

Other diketone homologs were synthesized following the same protocol from starting materials with respective chain lengths.

#### C_21_ 2-alkanol (2-heneicosanol)

In total, 91 mg of LiAlH_4_ were added to a solution of 500 mg 2-heneicosanone in 10 ml CH_2_Cl_2_. The reaction was quenched after 1 h by slowly adding 2 ml of 1 N HCl, and products were recovered by extraction with CHCl_3_. Organic fractions were combined, washed with saturated aqueous NaH_2_CO_3_, dried with Na_2_SO_4_, and concentrated under vacuum. The final product was purified by column chromatography (CHCl_3_).

#### C_14_–C_18_ 3-ketoacids

C_16_ 3-ketoacid methyl ester was synthesized as described previously^63^, with small modifications: 0.45 g myristic acid was dissolved in 3 ml CH_2_Cl_2_ containing 0.4 g N,N’-dicyclohexylcarbodiimide. Separately, 1.9 g 4-dimethylaminopyridine was added to 20 ml pyridine containing 1.6 g Meldrum’s acid. Both mixtures were stirred at room temperature for 15 min before combining them. After overnight stirring, the solvent was evaporated under vacuum and the solid re-dissolved in 20 ml methanol supplemented with two drops of concentrated H_2_SO_4_. The mixture was refluxed overnight, and product was extracted with Et_2_O. The combined organic fractions were washed with aqueous NaHCO_3_ solution, distilled water and finally saturated NaCl, the organic phase removed and dried with Na_2_SO_4_, and the solvent evaporated in vacuo. The resulting solid was purified by column chromatography (CHCl_3_) and product purity verified by GC-MS analysis.

In all, 0.5 g C_16_ 3-ketoacid methyl ester was dissolved in glacial acetic acid supplemented with concentrated HCl, and the mixture stored at 10 °C. Prior to each experiment, product was obtained by extraction with Et_2_O. The organic phase was washed with deionized water, and the solvent evaporated under vacuum. GC-MS analysis showed that the crude product contained non-hydrolyzed C_16_ 3-ketoacid methyl ester and C_16_ 3-ketoacid as main products, accompanied by small amounts of the decarboxylation product, C_15_ 2-ketone. This mixture was separated by flash column chromatography (CHCl_3_), to yield pure C_16_ 3-ketoacid for use in biochemical assays (while C_16_ 3-ketoacid methyl ester was recovered for further hydrolysis).

The same protocols were used to synthesize C_14_, C_17_, and C_18_ 3-ketoacids.

### Enzyme assays

#### *E. coli* in vivo assay

Three independent *E. coli* BL21 (DE3) colonies each carrying pET28-TEVH or pET28-HvDMH were inoculated into 1 ml LB liquid medium with kanamycin and precultured at 37 °C overnight. Then, each broth was diluted 1:100 in 100 ml LB liquid medium supplemented with kanamycin, cultivated at 37 °C to OD_600_ ~0.6, supplemented with 0.5 mM isopropyl β-D-1-thiogalactopyranoside (IPTG) to induce the expression of recombinant protein, and incubated at 22 °C overnight. Then, the OD_600_ were measured for normalization against cell numbers and *E. coli* cells collected by centrifugation for lipid analysis.

#### Yeast in vivo assay

Three independent yeast colonies each of lines expressing pESC-Trp-GAL1::LACS1-MYC or pESC-Trp-GAL1::LACS1-MYC-GAL10::HvDMP-FLAG were inoculated into 2 ml appropriate liquid minimal medium supplemented with 2% glucose and incubated at 28 °C overnight. The precultures were then expanded to 30 ml and cultivated for 18 h. The resulting yeast cells were collected by centrifugation and transferred into liquid minimal medium supplemented with 2% galactose to induce the expression of target genes and cultivated at 28 °C for 16 h. Then, yeast cells were transferred to 30 ml liquid minimal medium based on phosphate buffer (20 mM Na_2_HPO_4_/NaH_2_PO_4_, 300 mM NaCl, pH 7.4) and containing 2% galactose. Enzyme substrates (Supplementary Table S5) dissolved in ethanol were added (0.22 mM final concentrations of fatty acids and 3-ketoacids), and the yeast cells were cultivated for another 24 h at 22 °C. Finally, the OD_600_ of the resulting yeast were measured for normalization against cell numbers, and cells were harvested by centrifugation for further product analysis.

#### Protein purification and in vitro assay

*E. coli* BL21 (DE3) colonies carrying pET28a-EcACP, pET28a-EcFabD, pET28a-MtFabH, pET28-HvDMH or pET28-HvDMP were inoculated into 5 ml LB liquid medium with kanamycin and precultured at 37 °C overnight. *E. coli* precultures were diluted 1:100 in LB liquid medium supplemented with kanamycin and cultivated at 37 °C to OD_600_ ~0.6, and 0.5 mM IPTG was added to induce expression of recombinant protein. After incubating at 16 °C overnight (*E. coli* expressing MtFabH were incubated at 37 °C for 3 h), *E. coli* cells were collected by centrifugation (10,000× *g*, 4 °C). All following steps of protein purification were performed using phosphate-based buffers (20 mM Na_2_HPO_4_/NaH_2_PO_4_, 300 mM NaCl with imidazole, pH 7.4) supplemented with 1% Triton X-100, and equilibration buffer was supplemented with 0.1 mM phenylmethylsulfonyl fluoride (PMSF) as protease inhibitor. *E. coli* pellets were resuspended in equilibration buffer and lysed by sonication at 4 °C. The recombinant EcACP, EcFabD, MtFabH, HvDMH, and HvDMP proteins were obtained by Ni-NTA affinity purification with HisPur Ni-NTA resin (Invitrogen) following the manufacturer’s protocol. Respective recombinant proteins were eluted in phosphate buffer and dialyzed three times for 7 h in dialysis buffer (0.1 M Na_2_HPO_4_/NaH_2_PO_4_, 2.5 mM dithiothreitol (DTT), 10 mM MgCl_2_, 0.025% Triton X-100, pH 7.4) at 4 °C. The purity of recombinant proteins was verified by SDS-PAGE, and the protein concentration was determined by protein assay using the Bradford method with Protein Assay Dye Reagent Concentrate (Bio-Rad). The obtained proteins were used immediately in enzyme assays.

HvDMH in vitro assays were performed as described previously^40,41^. In brief, recombinant EcACP (1.0 mg), EcFabD (100 µg) and MtFabH (100 µg) were coincubated in 2 ml 1,3-bis(tris(hydroxymethyl) methylamino propane (20 mM, pH 7.0) containing malonyl-CoA (0.2 mM) and C_14_ acyl-CoA (0.2 mM) at 37 °C for 5 h. 500 µg of HvDMH were added, and the reaction was incubated for an additional 2 h. The reaction was quenched by one drop of 2 mM HCl with tetracosane (3 µg) supplemented as an internal standard.

For HvDMP in vitro assays, 50 µg of purified recombinant protein was incubated in 3 ml 50 mM phosphate buffer (pH 7.4) containing 2.5 mM DTT, 10 mM MgCl_2_ and 0.025% Triton X-100 with the substrate combinations (Supplementary Table S6) at room temperature for 14 h with gentle shaking. The reaction was quenched by adding 200 µl 10% H_2_SO_4_, and the reaction products were extracted immediately for lipid analysis.

### Lipid analysis

#### Wax analysis

Flag leaf sheaths and spikes were excised from *H. vulgare* cv. Morex plants grown 85 d after germination. Surface waxes were extracted by submerging tissues twice for 30 s in CHCl_3_, extracts were combined, and the solvent was evaporated under gentle N_2_ flow. Samples were transferred into GC vials and derivatized with 10 µl bis-*N,O*-trimethylsilyltrifluoroacetamide (BSTFA) in 10 µl pyridine at 72 °C for 45 min. The reagents were evaporated under gentle N_2_ flow, and the product was dissolved in 200 µl CHCl_3_. The derivatized wax mixtures were then analyzed on a Gas Chromatography system (6890 N, Agilent) equipped with HP-1 capillary column (30 m × 0.32 mm i.d., 1-µm film thickness, Agilent) coupled with Mass Selective Detector (5793 N, Agilent) using cool-on-column injection and the oven program: 50 °C held for 2 min, raised by 40 °C min^−1^ to 200 °C and held for 2 min, raised by 3 °C min^−1^ to 320 °C and held for 30 min.

#### *E. coli* lipid analysis

*E. coli* pellets were washed with distilled water, collected by centrifugation and allowed to air-dry. Known quantities of C_21_ 2-ketone, C_21_ 2-alkanol and C_20_ 2-hydroxyacid methyl ester were added to each sample for quantification of respective compound classes, *E. coli* cells were resuspended in 1 ml methanol containing 0.5 M sulfuric acid and 2% (v/v) 2,2-dimethoxypropane, and incubated at 80 °C for 3 h. Then 2.5% NaCl solution (w/v) was added, and the mixture was extracted three times with hexane, and the combined organic phases were concentrated under N_2_ flow and derivatized as described above. The final products were dissolved in CHCl_3_ and analyzed by GC-MS as above, but with the following oven program: 50 °C held for 2 min, raised by 40 °C min^−1^ to 100 °C and held for 2 min, raised by 3 °C min^−1^ to 320 °C and held for 5 min. The correction factor for quantifying 3-hydroxyacids was experimentally determined using authentic C_16_ 3-hydroxyacid and C_20_ 2-hydroxyacid standards.

#### Yeast lipid analysis

Overall, 30 ml-yeast cultures were split into 25 ml for β-diketone analysis and 5 ml for fatty acid profiling, and cells were collected by centrifugation, washed with distilled water, and allowed to air-dry. For product analysis, C_35_ 14,16- diketone was supplemented to yeast pellets as internal standard. Yeast cells were resuspended in 3 ml distilled water and lysed with 0.5 mm glass beads (BioSpec) by vertexing. 2 ml saturated NaCl solution was added, and total lipids were extracted three times with CHCl_3_, the combined organic phases were dried with Na_2_SO_4_ and filtered, and the solvent was evaporated under vacuum. The resulting lipids were transferred into GC vials, derivatized, and analyzed by GC-MS as described for wax analysis. All diketones were quantified using *m/z* 100 selected-ion traces. For fatty acid profiling, yeast cells were resuspended in 1 ml methanol containing 0.5 M sulfuric acid and 2% (v/v) 2,2-dimethoxypropane. The transmethylation reaction was performed as described above, and products were extracted with hexane, derivatized as previously described, and analyzed by GC-MS as described for *E. coli* lipid analysis.

#### In vitro product analysis

Samples from HvDMH in vitro assay were extracted three times with hexane, and the combined organic phases were concentrated under N_2_, derivatized and analyzed by GC-MS as described for wax analysis. Samples of the HvDMP in vitro reaction mixture were prepared in the same way, but extracting twice with CHCl_3_ and drying under vacuum.

### Carbon isotope analysis

Wax was extracted from *H. vulgare* cv. Morex flag leaf sheaths as described above and fractionated by TLC (CHCl_3_: hexane (1:1); 0.25 mm layer thickness; Analtech). TLC bands were stained with primuline (1% in acetone) and visualized under UV light, target fractions were scratched from the plate and extracted with CHCl_3_, and fraction purities were verified by GC-MS analysis. For the two fractions containing esters/alkanes and β-diketones, compound-specific carbon isotope compositions were determined using GC-IRMS as described before^64^. In brief, a trace GC system equipped with Rxi-5ms column (30 m × 0.25 mm, film thickness 0.25 µm, Restek) and a programmable temperature vaporizing injector operated in solvent split mode was connected via a GC Isolink to a combustion furnace at 1000 °C and via a Conflo IV interface further to a DeltaVPlus isotope ratio mass spectrometer (Thermo Scientific). The CO_2_ reference gas was analyzed daily with a standard deviation of <0.06‰ for instrument stability and linearity. Six reference peaks of CO_2_ bracketed analyte peaks during the course of a GC-IRMS run; two were used for standardization, the rest were used to monitor stability. Samples were interspersed with an external standard (A6mix) obtained from Dr. A. Schimmelmann, Indiana University, Bloomington, containing 15 alkanes (C_16_ to C_30_) with δ^13^C values ranging from −33.34 to −26.15‰ in order to establish a multi-point correlation, with which data were normalized to the Vienna Pee Dee Belemnite (VPDB) carbon isotopic scale.

### Barley protoplast and tobacco leaf transient expression and microscopy

Two-week-old *H. vulgare* cv. Morex seedlings were used for protoplast preparation and transformation following the method described previously^65^. pGWB5-35Spro::HvDMH-GFP was used for protoplast transient expression. Transformed protoplasts were incubated for 16–18 h and imaged by an Olympus FV1000 multiphoton confocal laser scanning microscope equipped with 405 nm, 473 nm, and 559 nm lasers. Transient expression of pGWB6-35Spro::GFP-HvDMP and ER-specific marker p35S:HDEL-RFP (obtained from Drs. M. Schuetz and L. Samuels, UBC, Vancouver) were in *N. benthamiana* was performed as described previously^66^ and imaged with the same microscope. GFP and RFP were at 488 nm and 561 nm, respectively, and fluorescence of GFP, RFP, and chlorophyll was detected at 525 nm, 595–625 nm, and 660–750 nm, respectively. ImageJ 1.51r software was used for image processing.

### Statistics and reproducibility

Microsoft Excel (version 2108) and GraphPad Prism 10 software were used for statistical analysis. Two-tailed Student’s *t* tests were used to assess significant differences between samples in different assays or wax analyses. Details are provided in the captions of the corresponding figures. All experiments and assays were repeated independently two to three times with similar results.

### Reporting summary

Further information on research design is available in the Nature Portfolio Reporting Summary linked to this article.

### Supplementary information

Supplementary Information

Peer Review File

Reporting Summary

### Source data

Source Data

## Data Availability

The sequences of the genes used in this article can be found in NCBI GenBank or the Arabidopsis information resource (TAIR) under the following accession numbers: HvDMH (KU721941, MLOC_13397, Cer-q); HvDMP (KU721941, MLOC_59804, Cer-c); EcACP (U00096); EcFabD (U00096); MtFabH (AL123456); LACS1(AT2G47240). The authors declare that all the data supporting the findings of this study are available within this paper and its Supplementary Information file. Source data are provided with this paper.
