# Supplementary material for: Biosynthesis of barley wax β-diketones: a type-III polyketide synthase condensing two fatty acyl units
Source: Nat Commun. 2023 Nov 10;14:7284. doi: 10.1038/s41467-023-42917-9 (PMC10638390; doi:10.1038/s41467-023-42917-9)
Supplement: Supplementary file 1 — Supplementary Information [file 41467_2023_42917_MOESM1_ESM.pdf]

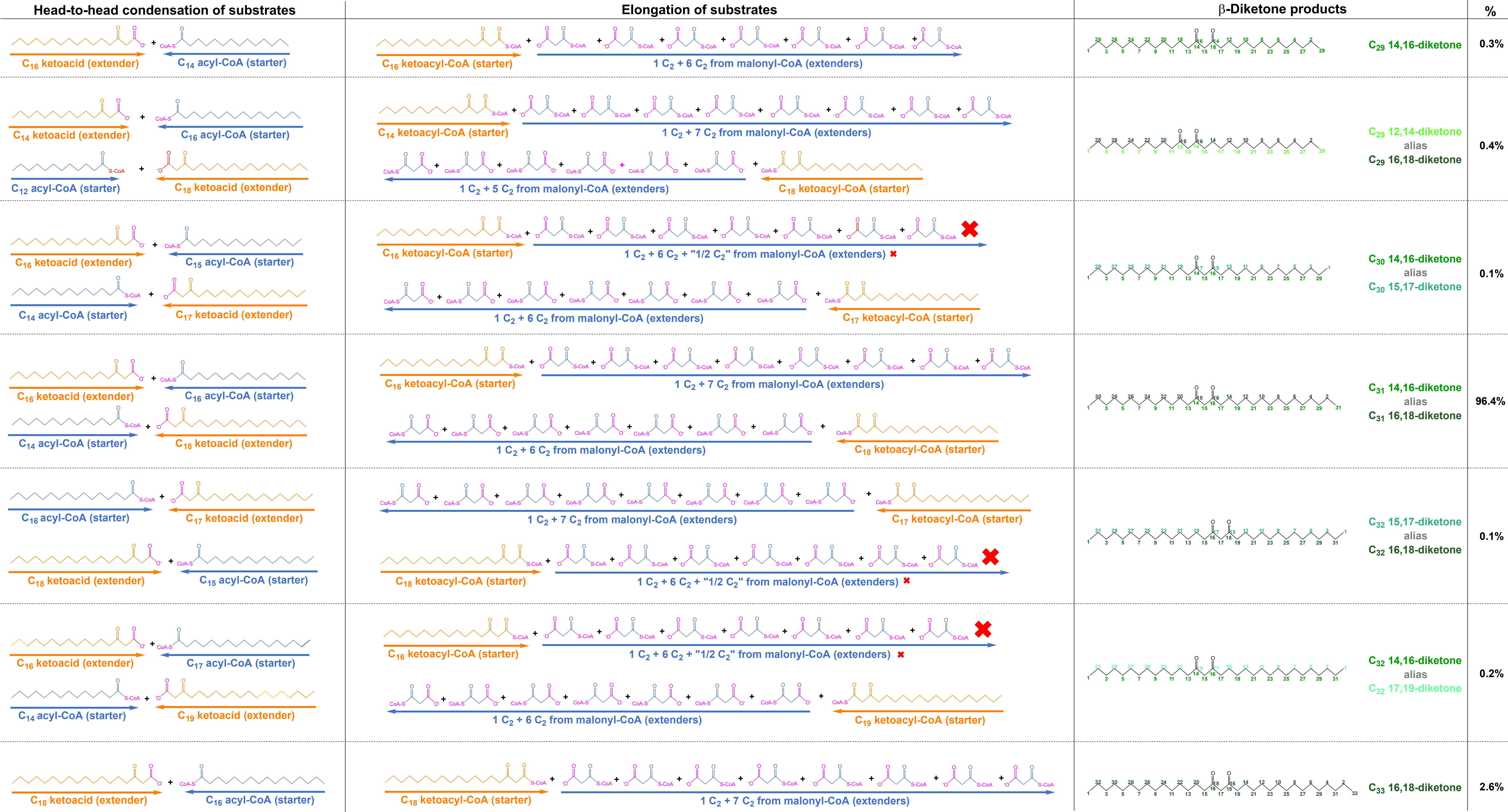

Head-to-head condensation of substrates
Elongation of substrates
β-Diketone products
%
C_{16} ketoacid (extender)
C_{14} acyl-CoA (starter)
C_{16} ketoacyl-CoA (starter)
1 C_2 + 6 C_2 from malonyl-CoA (extenders)
C_{29} 14,16-diketone
0.3%
C_{14} ketoacid (extender)
C_{16} acyl-CoA (starter)
C_{12} acyl-CoA (starter)
C_{18} ketoacid (extender)
C_{14} ketoacyl-CoA (starter)
1 C_2 + 7 C_2 from malonyl-CoA (extenders)
1 C_2 + 5 C_2 from malonyl-CoA (extenders)
C_{18} ketoacyl-CoA (starter)
C_{29} 12,14-diketone
alias
C_{29} 16,18-diketone
0.4%
C_{16} ketoacid (extender)
C_{15} acyl-CoA (starter)
C_{14} acyl-CoA (starter)
C_{17} ketoacid (extender)
C_{16} ketoacyl-CoA (starter)
1 C_2 + 6 C_2 + "1/2 C_2" from malonyl-CoA (extenders)
1 C_2 + 6 C_2 from malonyl-CoA (extenders)
C_{17} ketoacyl-CoA (starter)
C_{30} 14,16-diketone
alias
C_{30} 15,17-diketone
0.1%
C_{16} ketoacid (extender)
C_{16} acyl-CoA (starter)
C_{14} acyl-CoA (starter)
C_{18} ketoacid (extender)
C_{16} ketoacyl-CoA (starter)
1 C_2 + 7 C_2 from malonyl-CoA (extenders)
1 C_2 + 6 C_2 from malonyl-CoA (extenders)
C_{18} ketoacyl-CoA (starter)
C_{31} 14,16-diketone
alias
C_{31} 16,18-diketone
96.4%
C_{16} acyl-CoA (starter)
C_{17} ketoacid (extender)
C_{18} ketoacid (extender)
C_{15} acyl-CoA (starter)
1 C_2 + 7 C_2 from malonyl-CoA (extenders)
C_{17} ketoacyl-CoA (starter)
C_{18} ketoacyl-CoA (starter)
1 C_2 + 6 C_2 + "1/2 C_2" from malonyl-CoA (extenders)
C_{32} 15,17-diketone
alias
C_{32} 16,18-diketone
0.1%
C_{16} ketoacid (extender)
C_{17} acyl-CoA (starter)
C_{14} acyl-CoA (starter)
C_{19} ketoacid (extender)
C_{16} ketoacyl-CoA (starter)
1 C_2 + 6 C_2 + "1/2 C_2" from malonyl-CoA (extenders)
1 C_2 + 6 C_2 from malonyl-CoA (extenders)
C_{19} ketoacyl-CoA (starter)
C_{32} 14,16-diketone
alias
C_{32} 17,19-diketone
0.2%
C_{18} ketoacid (extender)
C_{16} acyl-CoA (starter)
C_{18} ketoacyl-CoA (starter)
1 C_2 + 7 C_2 from malonyl-CoA (extenders)
C_{33} 16,18-diketone
2.6%

Figure S1. Predicted building blocks for head-to-head condensation or elongation to individual β-diketone homologs and isomers.

Structures, names and relative amounts (%) of β-diketone products in barley *cv*. Morex spike wax are shown on the right. The building blocks required to form each product by head-to-head condensation are shown on the left, and building blocks required to form the products instead by elongation are shown in the center (compare Fig. 1). 3-Ketoacid intermediates are given in organe, and the fatty acyl-CoA or malonyl-CoAs for the two alternative synthesis mechanisms are given in blue. The groups labeled in magenta are lost during decarboxylative reactions. Blue and orange arrows below structures indicate the direction of chain formation. The malonyl-CoA extenders are counted as "1 C_2 + X C_2", to designate that the first C_2 unit is incorporated by HvDMP and "X" further C_2 units are incorporated by FAE complex(es). Two alternatives are shown where biosynthesis of a particular β-diketone product can occur in two directions. Red symbols "×" indicate that formation of a particular β-diketone product by elongation in that direction is impossible.

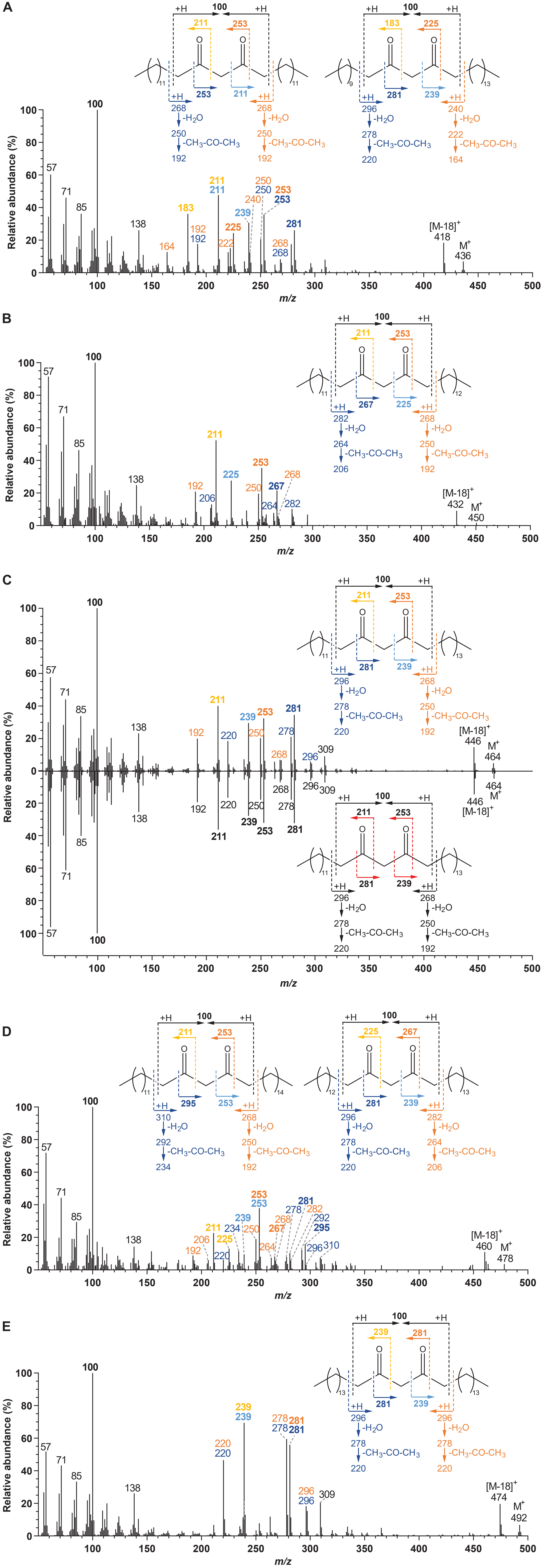
A
B
C
D
E
Relative abundance (%)
m/z
+H
100
-H2O
-CH3-CO-CH3
[M-18]+
418
M+
436
[M-18]+
432
M+
450
[M-18]+
446
M+
464
[M-18]+
460
M+
478
[M-18]+
474
M+
492

Figure S2. Mass spectra of β-diketone homologs/isomers identified in barley cv. Morex spike and flag leaf sheath waxes.

A) Mass spectrum of the mixture of wax C_{29} β-diketone isomers, and fragmentation patterns of C_{29} 14,16-diketone (left) and C_{29} 12,14-diketone (right). B) Mass spectrum and fragmentation pattern of wax C_{30} 14,16-diketone. C) Mass spectrum and fragmentation pattern of wax C_{31} 14,16-diketone (upper panel) and synthetic C_{31} 14,16-diketone (lower panel). D) Mass spectrum of the mixture of wax C_{32} β-diketone isomers, and fragmentation patterns of C_{32} 14,16-diketone (left) and C_{32} 15,17-diketone (right). E) Mass spectrum and fragmentation pattern of wax C_{33} 16,18-diketone.

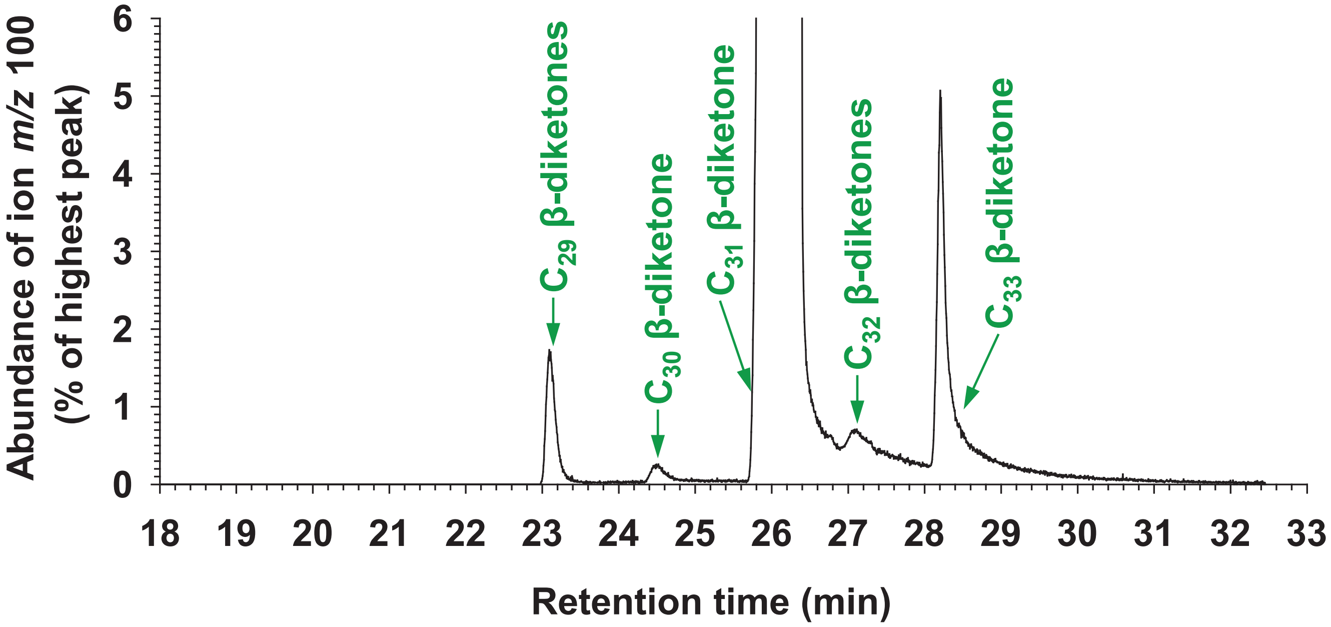

Figure S3. GC-MS analysis of the TLC fraction containing β-diketones from barley cv. Morex spike wax. The GC trace of the characteristic fragment *m/z* 100 shows five peaks of homologous β-diketones with equidistant retention times (retention time of each C_n diverging less than 2% from the average retention time differences between neighboring homologs C_{n-1} and C_{n+1}). This finding is in contrast with previous reports on various VLC compounds where, under GC conditions very similar to ours, skeletal isomers with methyl branches (*iso*-compounds) were separated from corresponding unbranched compounds by more than 25% of the homolog retention time difference[1]. Therefore, it can be ruled out that any of the β-diketones detected here had methyl branches.

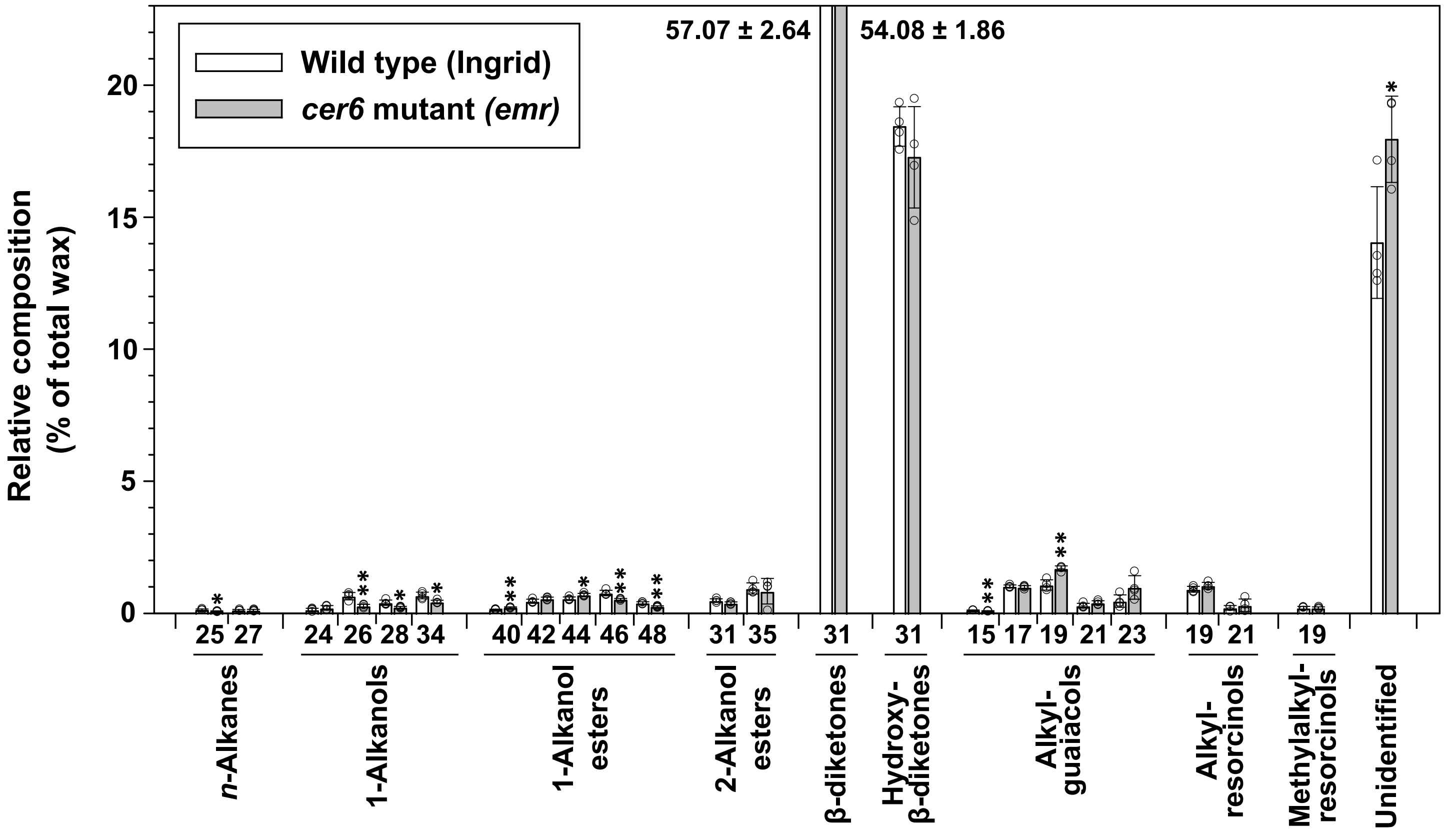

Figure S4. Wax analysis of a barley mutant deficient in elongation to very-long-chain compounds.

Numbers along the x-axis designate the chain lengths of compounds within each compound class. For alkylguaiacols (i.e., 1-methoxy-3-hydroxy-5-alkyl benzenes) and alkylresorcinols (i.e., 1,3-dihydroxy-5-alkyl-benzenes) carbon numbers in the alkyl side chains are given. Wax was extracted from flag leaf sheaths (at heading stage) of barley wild type (cv. Ingrid) and the *cer6* (*emr*) mutant known to have a defect in elongation of wax precursors beyond chain lengths C_{24}. GC-FID analysis confirmed the mutant phenotype, with amounts of $C_{>24}$ alkanes, 1-alkanols (primary alcohols) and esters containing these alkanols significantly reduced relative to wild type. In contrast, the amounts of β-diketones were not affected in the *cer6* (*emr*) mutant. C_{30} 1-alkanol and C_{33} 2-alkanol ester could not be separated from C_{21} alkylresorcinol under the present conditions and were, therefore, not quantified. Similarly, C_{32} 1-alkanol could not be separated from C_{31} β-diketone and was not quantified. Error bars represent standard deviations of four biological replicates. Asterisks indicate discovery of statistically significant differences between wild type and mutant, as calculated by two-tailed Student's *t*-test; $*P < 0.05$, $**P < 0.01$. The *P*-value between Ingrid and emr is: P (C_{25} n-alkane) = 0.0118; P (C_{26} 1-alkanol) = 0.0027; P (C_{28} 1-alkanol) = 0.0169; P (C_{34} 1-alkanol) = 0.0143; P (C_{40} 1-alkanol ester) = 0.0011; P (C_{44} 1-alkanol ester) = 0.0183; P (C_{46} 1-alkanol ester) = 0.0045; P (C_{48} 1-alkanol ester) = 0.0018; P (C_{15} alkylguaiacol) = 0.0026; P (C_{19} alkylguaiacol) = 0.0012; P (Unidentified) = 0.0263.

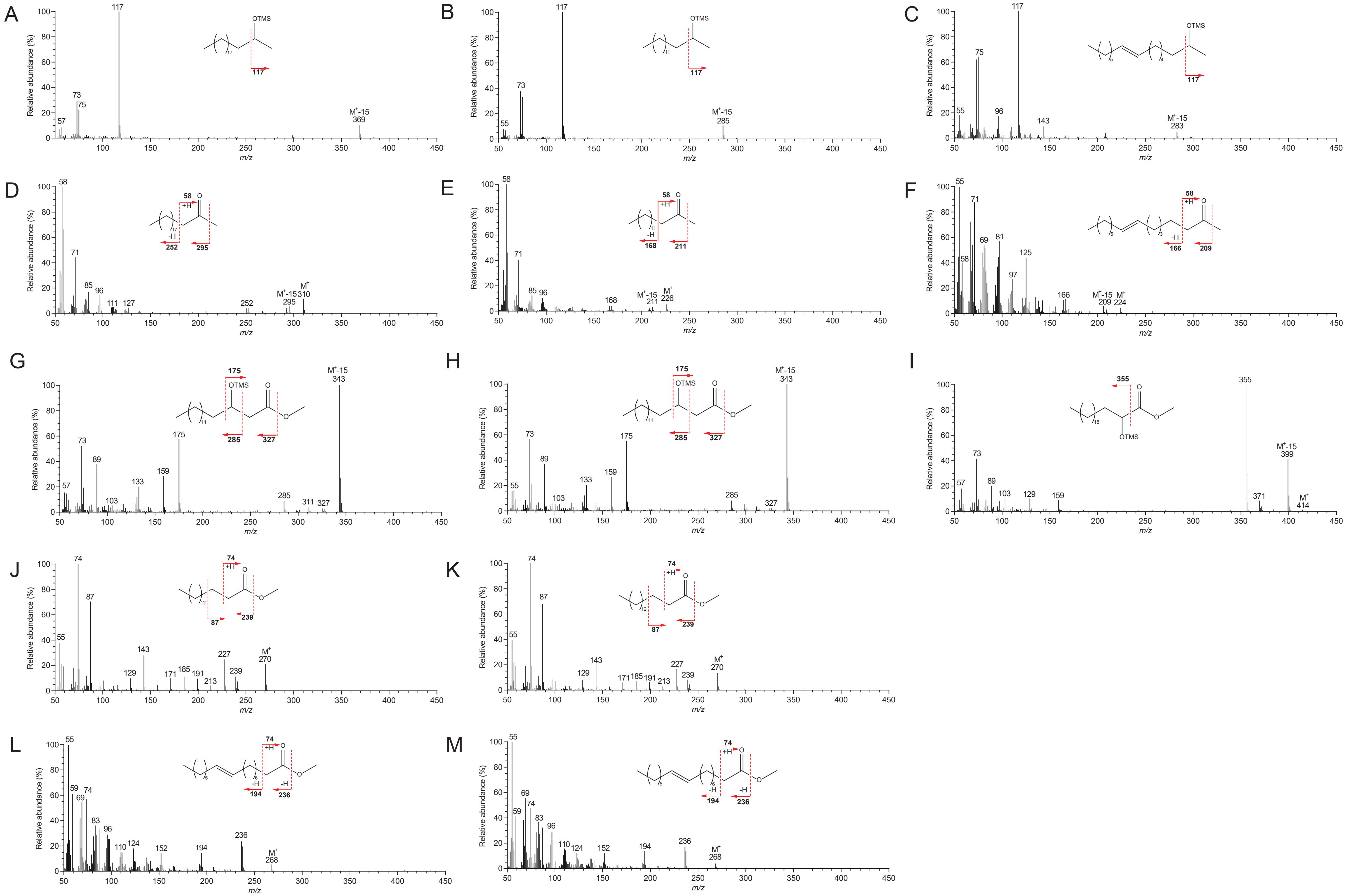

A
117
OTMS
17
117
M+-15
369
57
73
75
Relative abundance (%)
m/z
B
117
OTMS
11
117
73
55
M+-15
285
C
117
75
OTMS
5
4
117
55
96
143
M+-15
283
D
58
71
85
96
111
127
252
M+-15
295
M+
310
58
+H
-H
17
252
295
E
58
71
85
96
168
M+-15
211
M+
226
58
+H
-H
11
168
211
F
55
71
69
81
58
97
125
166
M+-15
209
M+
224
58
+H
-H
5
3
166
209
G
175
OTMS
11
285
327
M+-15
343
73
89
57
103
133
159
175
285
311
327
H
175
OTMS
11
285
327
M+-15
343
73
89
55
103
133
159
175
285
327
I
355
16
OTMS
355
M+-15
399
73
57
89
103
129
159
371
M+
414
J
74
+H
12
87
239
74
87
55
129
143
171
185
191
213
227
239
M+
270
K
74
+H
12
87
239
74
87
55
129
143
171
185
191
213
227
239
M+
270
L
74
+H
5
6
-H
-H
194
236
55
59
69
74
83
96
110
124
152
194
236
M+
268
M
74
+H
5
6
-H
-H
194
236
55
59
69
74
83
96
110
124
152
194
236
M+
268

Figure S5. MS analysis of acyls produced in *E. coli* expressing the barley Diketone Metabolism Hydrolase (HvDMH) enzyme.

A) Mass spectrum and fragmentation pattern of (saturated) C_{21} 2-alkanol standard. B) Mass spectrum and fragmentation pattern of (saturated) C_{15} 2-alkanol produced by *E. coli* expressing *HvDMH*. C) Mass spectrum and fragmentation pattern of monounsaturated C_{15} 2-alkanol produced by *E. coli* expressing *HvDMH*. D) Mass spectrum and fragmentation pattern of (saturated) C_{21} 2-ketone standard. E) Mass spectrum and fragmentation pattern of (saturated) C_{15} 2-ketone produced by *E. coli* expressing *HvDMH*. F) Mass spectrum and fragmentation pattern of monounsaturated C_{15} 2-ketone produced by *E. coli* expressing *HvDMH*. G) Mass spectrum and fragmentation pattern of (saturated) C_{16} 3-hydroxyacid methyl ester (ME) standard. H) Mass spectrum and fragmentation pattern of (saturated) C_{16} 3-hydroxyacid ME extracted from *E. coli* expressing *HvDMH*. I) Mass spectrum and fragmentation pattern of (saturated) C_{20} 2-hydroxyacid ME standard. J) Mass spectrum and fragmentation pattern of (saturated) C_{16} fatty acid ME standard. K) Mass spectrum and fragmentation pattern of (saturated) C_{16} fatty acid ME extracted from *E. coli* expressing *HvDMH*. L) Mass spectrum and fragmentation pattern of monounsaturated C_{16} fatty acid ME standard. M) Mass spectrum and fragmentation pattern of monounsaturated C_{16} fatty acid ME extracted from *E. coli* expressing *HvDMH*.

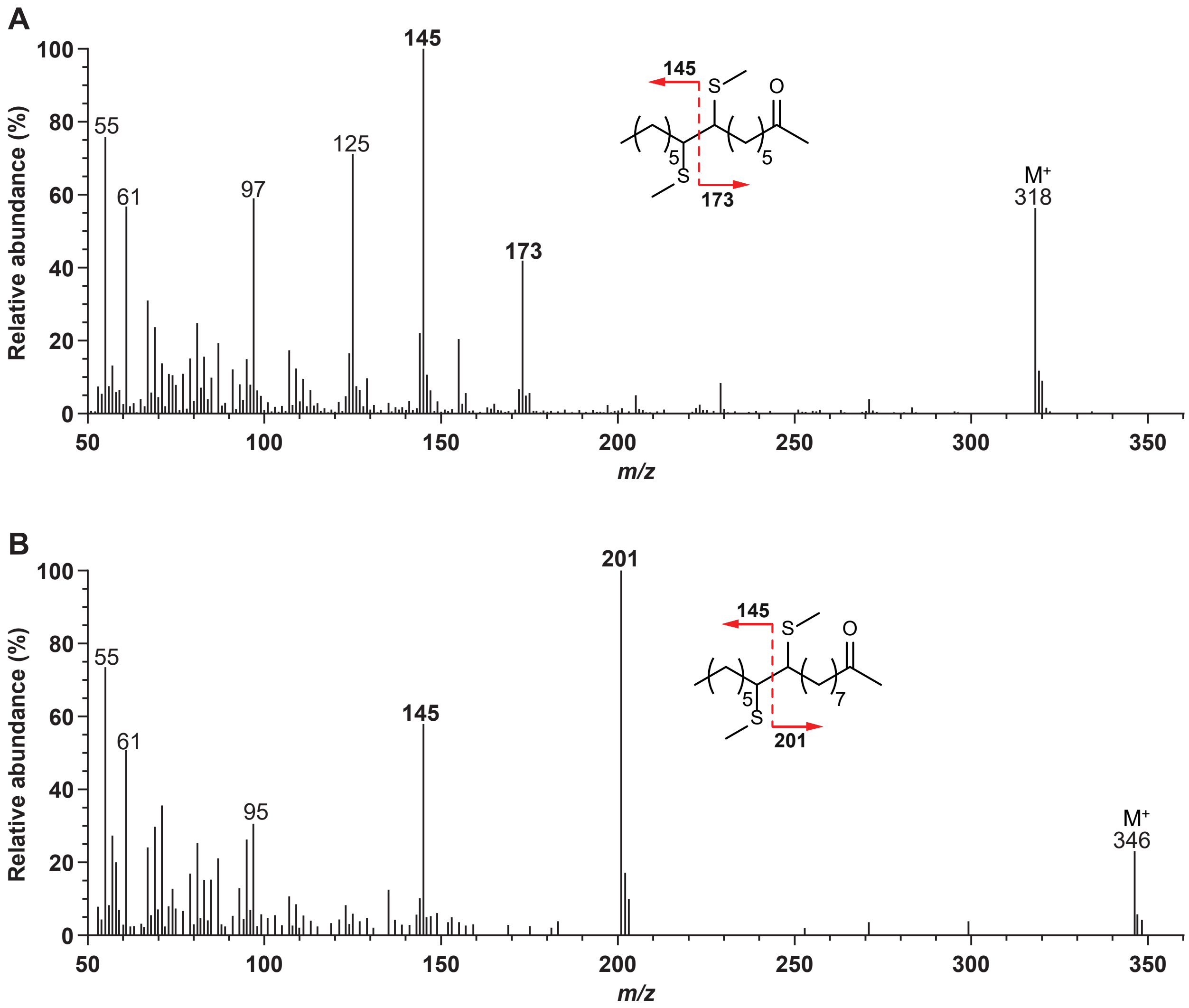

Figure S6. MS analysis of double-bond positions in monounsaturated 2-ketones produced in *E. coli* expressing the barley Diketone Metabolism Hydrolase (HvDMH) enzyme.

A) Mass spectrum of the dimethyldisulfide (DMDS) adduct of monounsaturated C_{15} 2-ketone produced by *E. coli* expressing *HvDMH*. B) Mass spectrum of the DMDS adduct of monounsaturated C_{17} 2-ketone produced by *E. coli* expressing *HvDMH*. The structure inserts show major fragmentation reactions. Both compounds show characteristic α-fragments *m/z* 145, revealing that the double bonds in the original 2-ketones were located seven carbons away from the methyl terminus. The 2-ketones are thus identified as ω-7 isomers.

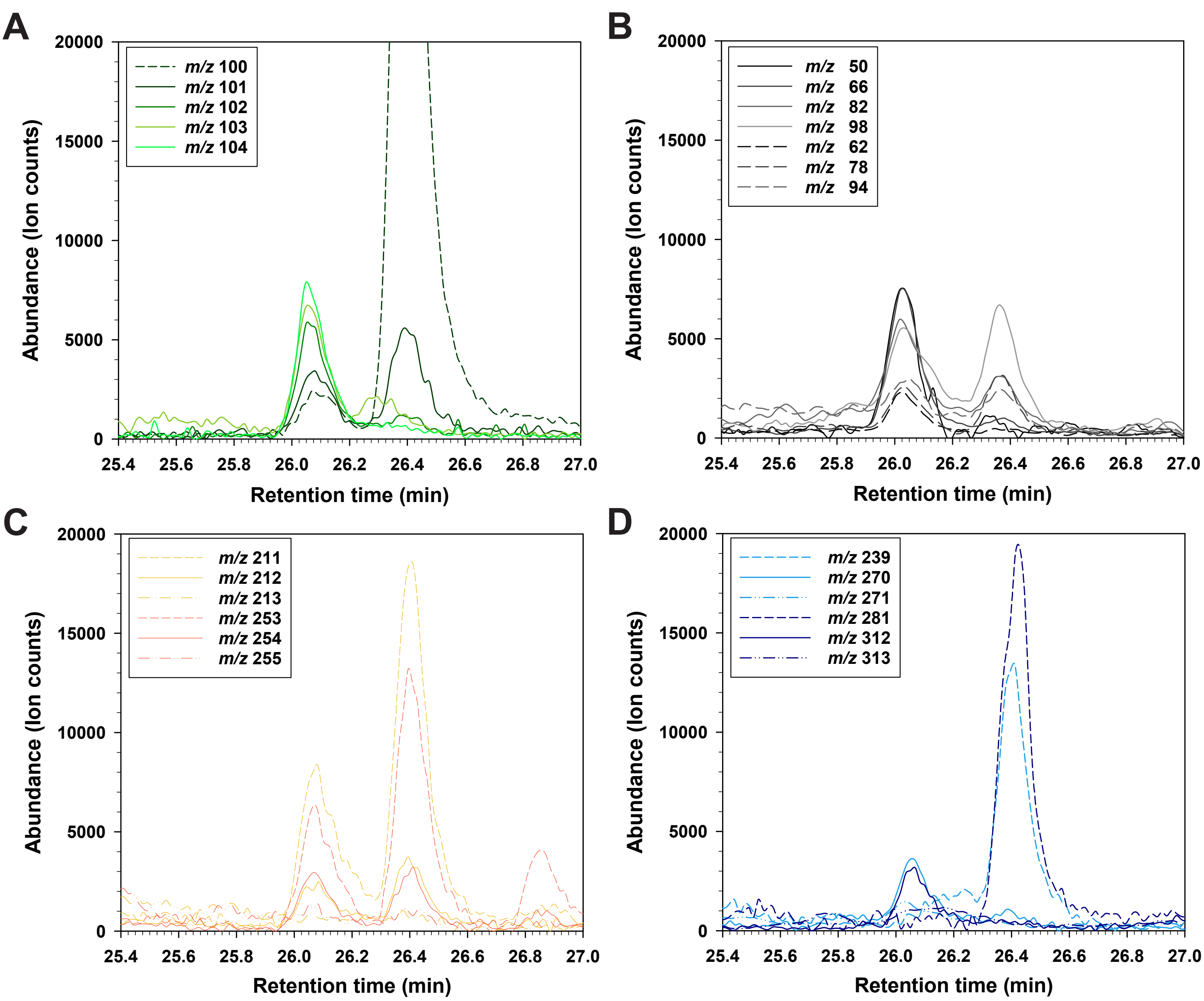

Figure S7. GC-MS analysis of deuterium-labeled β-diketone products from yeast in vivo assay.

Selected-ion chromatograms of characteristic fragments of D_{31}-labelled C_{31} 14,16-diketone produced in yeast expressing *HvDMP* and supplemented with C_{16} 3-ketoacid and per-deuterated fatty acid $C_{15}D_{31}COOH$. A) Traces of ion *m/z* 100 characteristic of the undeuterated β-diketo group and of ions *m/z* 101-104 characteristic of the deuterated β-diketo group. B) Traces of small-mass ions further characterizing the deuterated β-diketone product. It is plausible that the fragments *m/z* 50, 66, 82 and 98 have structures $[CD_3\text{-}(CD_2)_n]^+$ with n = 2-5, indicating alkyl decay of the perdeuterated chain terminus. The fragments *m/z* 62, 78 and 94 may be tentatively interpreted as $[CD_3\text{-}(CD_2)_n\text{-}CO]^+$ with n = 2-4. C) Traces of two α-fragments (*m/z* 211 and 253) characteristic of one undeuterated hydrocarbon tail of the β-diketone and of two corresponding deuterated fragments (*m/z* 212/213 and 254/255). D) Traces of two α-fragments (*m/z* 239 and 281) characteristic of the other undeuterated hydrocarbon tail of the β-diketone and of two corresponding perdeuterated fragments (*m/z* 270/271 and 312/313).

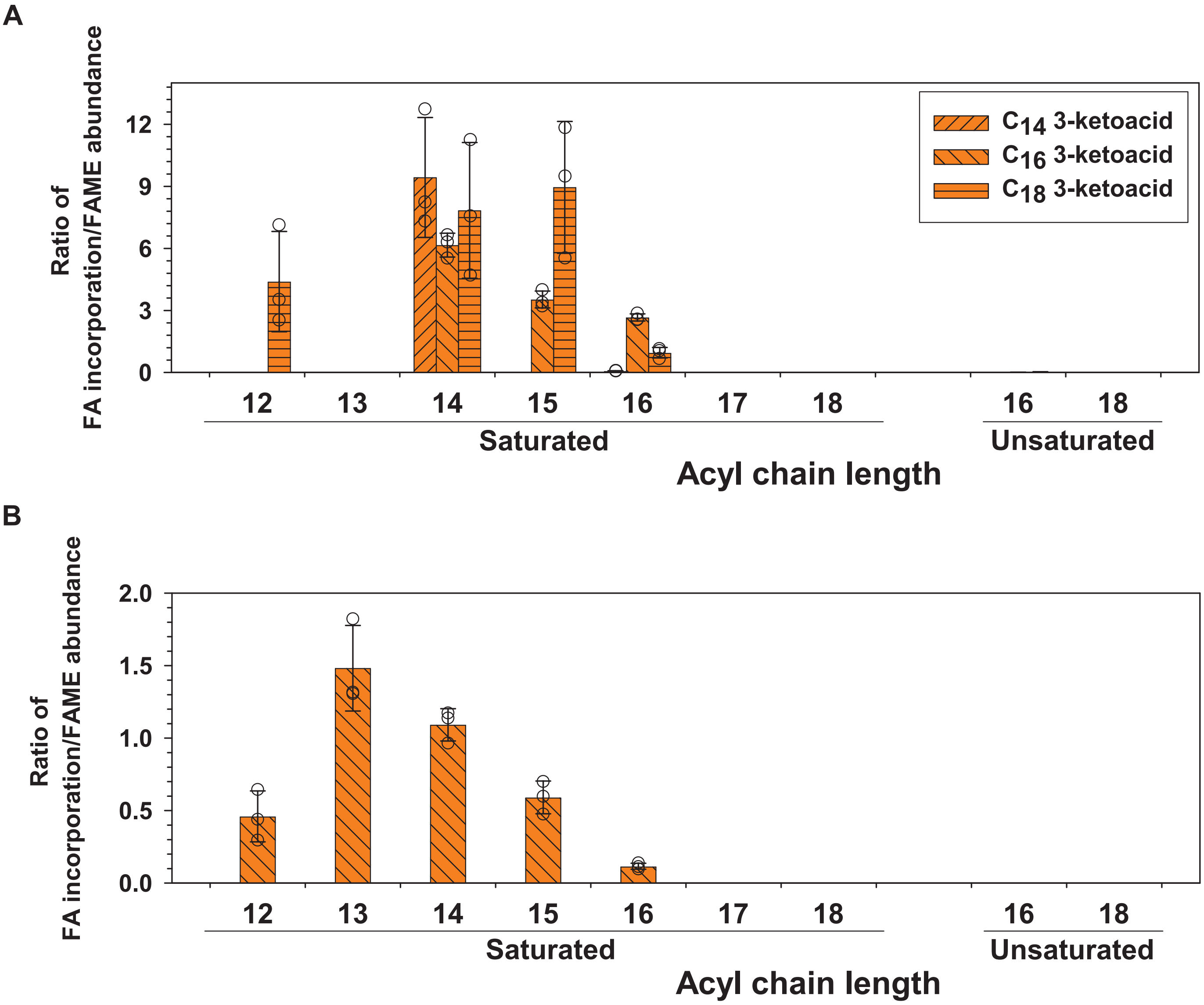

Figure S8. Determination of HvDMP substrate preferences by in vivo assays.

A) Ratios of fatty acyl substrate amount incorporated into particular β-diketones (together with a given 3-ketoacid co-substrate) to the amount of the corresponding fatty acyl available in yeast under the assay conditions (calculated from data in Fig. 6 A-C). B) Ratios of fatty acyl substrate amount incorporated into particular β-diketones (together with C_{16} ketoacid co-substrate) to the amount of the corresponding fatty acyl available in yeast complemented with C_{12}-C_{15} fatty acids (calculated from data in Fig. 6 D-F). Arabidopsis *LACS1* was expressed in all the yeast in vivo assays to enhance exogenous substrate uptake[2]. Error bars represent standard deviations of three biological replicates.

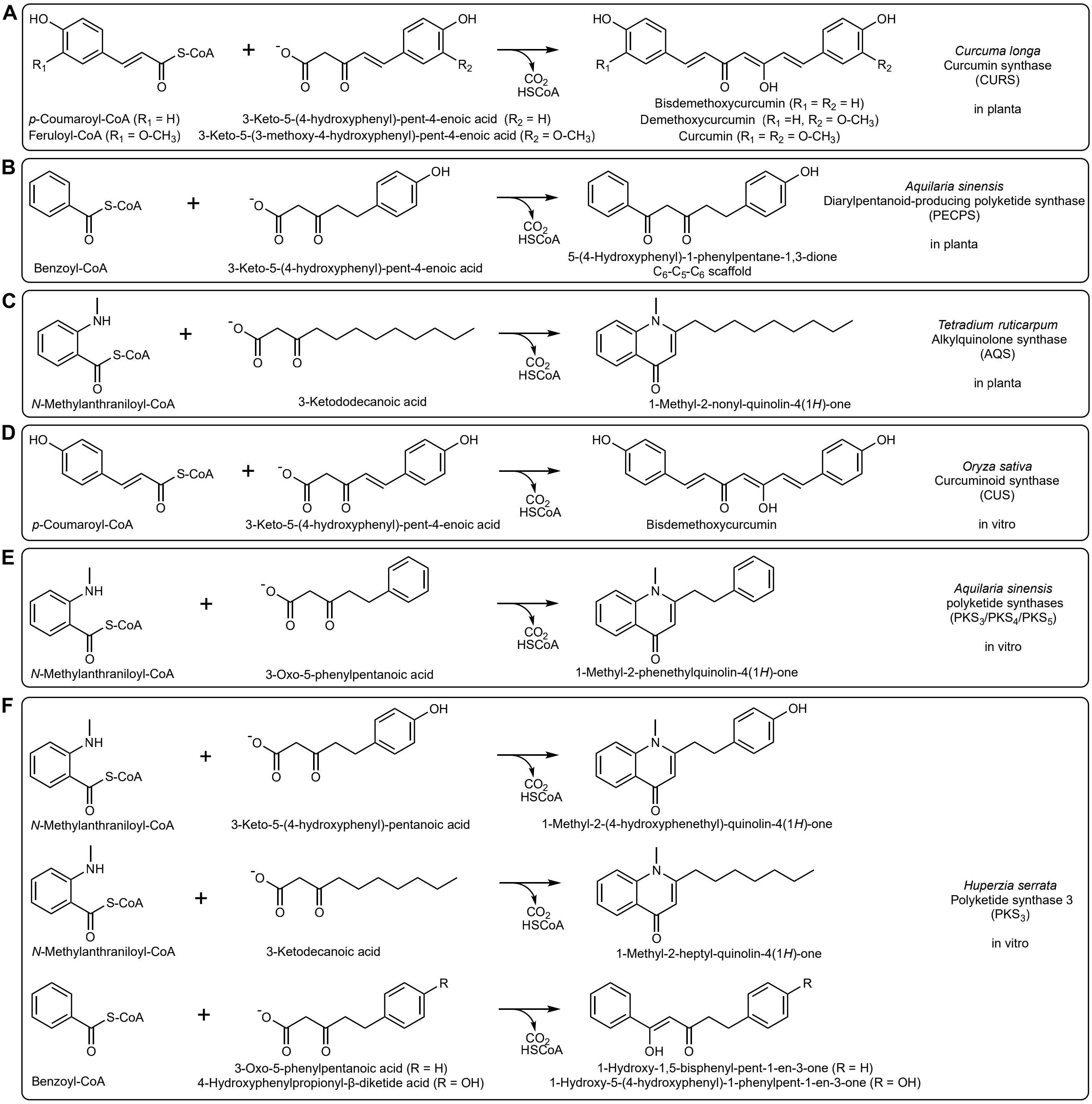

Figure S9. Reactions catalyzed by non-canonical plant type-III PKSs.

In planta or in vitro head-to-head condensation reactions catalyzed by A) *Curcuma longa* curcumin synthases (CURSs)[3,4], B) *Aquilaria sinensis* phenylethylchromone-forming polyketide synthase (PECPS)[5], C) *Tetradium ruticarpum* alkylquinolone synthase (AQS)[6], D) *Oryza sativa* curcuminoid synthase (CUS)[7,8], E) *Aquilaria sinensis* polyketide synthases (PKS3/PKS4/PKS5)[9] and F) *Huperzia serrata* polyketide synthase 3 (PKS3)[10].

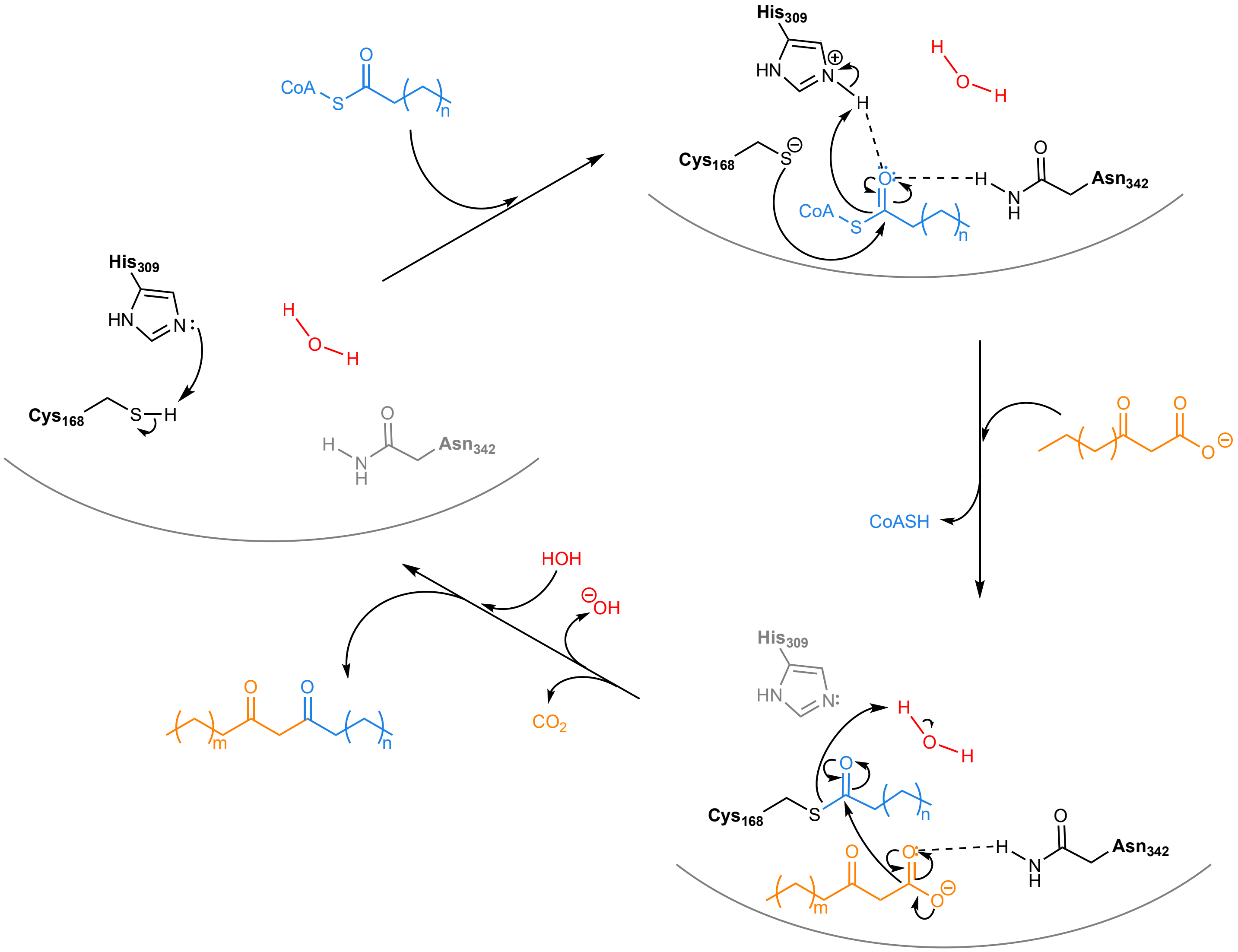

Figure S10. Proposed HvDMP reaction mechanism.

On the left, the empty enzyme active site is shown with the triad of amino acids predicted to participate in the mechanism. In the first step of the reaction cycle, a fatty acyl-CoA starter molecule is loaded into the catalytic center and covalently bound to Cys_{168}. Hydrogen bonds between the fatty acyl-CoA head group and His_{309} and Asn_{342} facilitate the bond formation. In the second step of the catalytic cycle, a 3-ketoacid extender molecule enters the active site and, assisted by Asn_{342}, undergoes decarboxylation into the corresponding enolate. Nucleophilic attack of the enolate on the carbonyl carbon of the starter and proton transfer via an adjacent water molecule (likely assisted by further amino acids lining the active site) yield the β-diketone product and re-set the active site.

Supplementary Table S1. Homolog and isomer distribution of β-diketones in barley *cv.* Morex sheath wax (% of compound class).

Keto group position \ β-Diketone chain length	C_{29}			C_{30}			C_{31}			C_{32}			C_{33}		
12,14	0.33	±	0.04	0.00	±	0.00	0.00	±	0.00	0.00	±	0.00	0.00	±	0.00
14,16	0.13	±	0.03	0.16	±	0.03	98.98	±	0.12	0.04	±	0.01	0.00	±	0.00
15,17	0.00	±	0.00	0.00	±	0.00	0.00	±	0.00	0.01	±	0.01	0.00	±	0.00
16,18	0.00	±	0.00	0.00	±	0.00	0.00	±	0.00	0.00	±	0.00	0.35	±	0.04

Supplementary Table S2. Homolog and isomer distribution of 2-alkanol esters in barley *cv.* Morex spike wax (% of compound class).

Esterified 2-alkanol \ Ester chain length	C_{33}	C_{34}	C_{35}	C_{36}	C_{37}
C_{13}	0.98 ± 0.39	0.00 ± 0.00	0.18 ± 0.18	0.00 ± 0.00	0.00 ± 0.00
C_{15}	7.27 ± 0.93	0.00 ± 0.00	56.29 ± 3.79	3.03 ± 0.15	17.90 ± 3.04
C_{17}	0.00 ± 0.00	0.00 ± 0.00	1.99 ± 0.89	0.00 ± 0.00	12.36 ± 1.68

Supplementary Table S3. Homolog and isomer distribution of 2-alkanol esters in barley *cv.* Morex sheath wax (% of compound class).

Esterified 2-alkanol \ Ester chain length	C_{33}	C_{34}	C_{35}	C_{36}	C_{37}
C_{13}	1.01 ± 0.28	0.00 ± 0.00	0.12 ± 0.21	0.00 ± 0.00	0.00 ± 0.00
C_{15}	7.25 ± 1.16	0.00 ± 0.00	57.51 ± 3.93	3.03 ± 0.15	22.68 ± 4.97
C_{17}	0.00 ± 0.00	0.00 ± 0.00	0.83 ± 0.54	0.00 ± 0.00	7.57 ± 0.32

Supplementary Table S4. Primers used in this study.

Construct	Primer name	Primer sequence
pET28a-EcACP	EcACP-NdeI-F	5'-CGGTTAGCATATGATGAGCACTATCGAAGAACGCG-3'
	EcACP-BamHI-R	5'-TTGGATCCTTACGCCTGGTGGCCGTTGAT-3'
pET28a-EcFabD	EcFabD-NdeI-F	5'-GCAATTCCATATGATGACGCAATTTGCATTTGTGT-3'
	EcFabD-BamHI-R	5'-CGGGATCCTTTTAAAGCTCGAGCGC-3'
pET28a-MtFabH	MtFabH-NdeI-F	5'-GCAATTCCATATGATGACGGAGATCGCCACGAC-3'
	MtFabH-BamHI-R	5'-ATGGATCCTCAACCCTTCGGCATTCGCA-3'
pGWB5-35Spro::HvDMH-GFP	HvDMH-F	5’-ATGCCTGCAAACAAGACTTAC-3’
	HvDMH-R	5’-GAAACAGTTGTTCATCATGGATC-3’
pGWB6-35Spro::GFP-HvDMP	HvDMP-F	5’-ATGGCAGGCAGCTCACC-3’
	HvDMP-R	5’-TTTTTTCTTGAGAGCGCCGG-3’
pESC-Trp-GAL1::LACS1-MYC	LACS1-BamHI-F	5’-AGAGGATCCATGAAGTCTTTTGCGGCTAAG-3’
	LACS1-SalI-R	5’-AGAGTCGACTGAGATTTTCTTTGAGGCCAAT-3’
pESC-Trp-GAL1::LACS1-MYC-GAL10::HvDMP-FLAG	HvDMP-EcoRI-F	5’-TCTGAATTCATGGCAGGCAGCTCACC-3’
	HvDMP-SpeI-R	5’-GGACTAGTAGCCATTTTTTCTTGAGAGCGC-3’

Supplementary Table S5. Substrates used to supplement HvDMP yeast in vivo assays.

Assay purpose	Fatty acyl substrate	3-Ketoacid substrate
Enzyme function	-	0.22 mM C_{16} 3-ketoacid
Deuterium-labeling	0.22 mM per-deuterated C_{16} fatty acid	0.22 mM C_{16} 3-ketoacid
Odd-numbered substrate labeling	0.22 mM C_{15} fatty acid	0.22 mM C_{16} 3-ketoacid
	0.22 mM C_{14} fatty acid	0.22 mM C_{17} 3-ketoacid
Substrate preference	-	0.22 mM C_{14} 3-ketoacid<br>0.22 mM C_{16} 3-ketoacid<br>0.22 mM C_{18} 3-ketoacid
	0.66 mM C_{12} fatty acid<br>0.66 mM C_{13} fatty acid<br>0.33 mM C_{14} fatty acid<br>0.33 mM C_{15} fatty acid	0.22 mM C_{16} 3-ketoacid

Supplementary Table S6. Substrate combinations used in HvDMP in vitro assays.

Assay purpose	Substrate I	Substrate II	Substrate III	Substrate IV
Enzyme function	0.15 mM C_{16} acyl-CoA lithium salt	0.15 mM C_{16} 3-ketoacid	-	-
	0.15~0.45 mM malonyl-CoA lithium salt	0.15 mM C_{16} 3-ketoacid	-	-
	0.15~0.45 mM malonyl-CoA lithium salt	0.15 mM C_{16} acyl-CoA lithium salt	-	-
Fatty acyl-CoA preference (competition assays)	0.15 mM C_{14} acyl-CoA lithium salt	0.15 mM C_{16} acyl-CoA lithium salt	0.15 mM C_{18} acyl-CoA lithium salt	0.225 mM C_{14} 3-ketoacid
	0.15 mM C_{14} acyl-CoA lithium salt	0.15 mM C_{16} acyl-CoA lithium salt	0.15 mM C_{18} acyl-CoA lithium salt	0.225 mM C_{16} 3-ketoacid
	0.15 mM C_{14} acyl-CoA lithium salt	0.15 mM C_{16} acyl-CoA lithium salt	0.15 mM C_{18} acyl-CoA lithium salt	0.225 mM C_{18} 3-ketoacid
3-Ketoacid preference (competition assays)	0.15 mM C_{14} 3-ketoacid	0.15 mM C_{16} 3-ketoacid	0.15 mM C_{18} 3-ketoacid	0.225 mM C_{14} acyl-CoA lithium salt
	0.15 mM C_{14} 3-ketoacid	0.15 mM C_{16} 3-ketoacid	0.15 mM C_{18} 3-ketoacid	0.225 mM C_{16} acyl-CoA lithium salt
	0.15 mM C_{14} 3-ketoacid	0.15 mM C_{16} 3-ketoacid	0.15 mM C_{18} 3-ketoacid	0.225 mM C_{18} acyl-CoA lithium salt

Supplementary References

1. Busta, L. & Jetter, R. Structure and biosynthesis of branched wax compounds on wild type and wax biosynthesis mutants of *Arabidopsis thaliana*. *Plant Cell Physiol.* **58**, 1059-1074 (2017).

2. Pulsifer, I. P. Kluge, S. & Rowland, O. Arabidopsis LONG-CHAIN ACYL-COA SYNTHETASE 1 (LACS1), LACS2, and LACS3 facilitate fatty acid uptake in yeast. *Plant Physiol. Biochem.* **51**, 31-39 (2012).

3. Katsuyama, Y. et al. Curcuminoid biosynthesis by two type III polyketide synthases in the herb *Curcuma longa*. *J. Biol. Chem.* **284**, 11160-11170 (2009).

4. Katsuyama, Y. Kita, T. & Horinouchi, S. Identification and characterization of multiple curcumin synthases from the herb *Curcuma longa*. *FEBS Lett.* **583**, 2799-2803 (2009).

5. Wang, X.-H. et al. Identification of a diarylpentanoid-producing polyketide synthase revealing an unusual biosynthetic pathway of 2-(2-phenylethyl) chromones in agarwood. *Nat. Commun.* **13**, 348 (2022).

6. Matsui, T. et al. 2-Alkylquinolone alkaloid biosynthesis in the medicinal plant *Evodia rutaecarpa* involves collaboration of two novel type III polyketide synthases. *J. Biol. Chem.* **292**, 9117-9135 (2017).

7. Katsuyama, Y. et al. *In vitro* synthesis of curcuminoids by type III polyketide synthase from *Oryza sativa*. *J. Biol. Chem.* **282**, 37702-37709 (2007).

8. Morita, H. et al. Structural basis for the one-pot formation of the diarylheptanoid scaffold by curcuminoid synthase from *Oryza sativa*. *Proc. Natl. Acad. Sci. USA* **107**, 19778-19783 (2010).

9. Xiao, M. et al. Three candidate 2-(2-phenylethyl) chromone-producing type III polyketide synthases from *Aquilaria sinensis* (Lour.) Gilg have multifunctions synthesizing benzalacetones, quinolones and pyrones. *Ind. Crops Products* **186**, 115263 (2022).

10. Wang, J. et al. Synthesis of unnatural 2-substituted quinolones and 1,3-diketones by a member of type III polyketide synthases from *Huperzia serrata*. *Org. Lett.* **18**, 3550-3553 (2016).
